# *De Novo* Assembled Wheat Transcriptomes Delineate Differentially Expressed Host Genes in Response to Leaf Rust Infection

**DOI:** 10.1371/journal.pone.0148453

**Published:** 2016-02-03

**Authors:** Saket Chandra, Dharmendra Singh, Jyoti Pathak, Supriya Kumari, Manish Kumar, Raju Poddar, Harindra Singh Balyan, Puspendra Kumar Gupta, Kumble Vinod Prabhu, Kunal Mukhopadhyay

**Affiliations:** 1 Department of Bio-Engineering, Birla Institute of Technology, Mesra, Ranchi 835215 Jharkhand, India; 2 Department of Genetics and Plant Breeding, Ch. Charan Singh University, Meerut 200005, Uttar Pradesh, India; 3 Division of Genetics, Indian Agricultural Research Institute, New Delhi 110012, India; Nanjing Agricultural University, CHINA

## Abstract

Pathogens like *Puccinia triticina*, the causal organism for leaf rust, extensively damages wheat production. The interaction at molecular level between wheat and the pathogen is complex and less explored. The pathogen induced response was characterized using mock- or pathogen inoculated near-isogenic wheat lines (with or without seedling leaf rust resistance gene *Lr28*). Four Serial Analysis of Gene Expression libraries were prepared from mock- and pathogen inoculated plants and were subjected to Sequencing by Oligonucleotide Ligation and Detection, which generated a total of 165,767,777 reads, each 35 bases long. The reads were processed and multiple k-mers were attempted for *de novo* transcript assembly; 22 k-mers showed the best results. Altogether 21,345 contigs were generated and functionally characterized by gene ontology annotation, mining for transcription factors and resistance genes. Expression analysis among the four libraries showed extensive alterations in the transcriptome in response to pathogen infection, reflecting reorganizations in major biological processes and metabolic pathways. Role of auxin in determining pathogenesis in susceptible and resistant lines were imperative. The qPCR expression study of four LRR-RLK (Leucine-rich repeat receptor-like protein kinases) genes showed higher expression at 24 hrs after inoculation with pathogen. In summary, the conceptual model of induced resistance in wheat contributes insights on defense responses and imparts knowledge of *Puccinia triticina*-induced defense transcripts in wheat plants.

## Introduction

The advent of high-throughput next-generation sequencing (NGS) technologies in the past decade resulted in massive drop in cost of sequencing-per-base that steered the expansion of its applications to a wide range of organisms. This contributed towards the discovery and annotation of millions of genes in several model organisms. However, the progress of genome sequencing has been slow in non-model organisms with large genomes. Bread wheat (*Triticum aestivum* L., 2*n* = 6x = 42) is one of the most important food crops in the world. But sequencing of wheat genome was taken up rather late due to several factors including its large genome size (16.94 Gb), extensive abundance of repetitive elements (~80%) [1] and high levels of methylation and transposition in the intergenic regions [2]. Moreover, recent polyploidization from closely related progenitors complicates alignment of homoeologous sequences belonging to its three sub-genomes [3]. It is also perceived that only 1–2% of the wheat genome is transcribed and translated into proteins [4]. Only recently, a low (5x) coverage and the chromosome arm based draft genome sequence of wheat became available [5, 6]. Therefore, in the absence of a completely sequenced genome, transcriptome sequencing has been considered to be an effective alternative for rapid identification of wheat genes [7, 8]. NGS of transcriptomes provide extensive data in a short period with high depth and coverage that can be utilized for gene discovery, identification of SNPs and other functional markers. The data can also be utilized for a comparative study of transcriptomes under various physiological conditions leading to altered metabolic processes [9, 10]. *De novo* assembled transcriptomes have been important sources for identification of functional genes involved in different metabolic pathways from non-model plants like Eucalyptus [11], rubber plant [12] and chickpea [9]. Candidate genes for salt tolerance from *Gossypium aridum* [13] and genes presumably involved in flowering in *Fagopyrum* [14] were identified through comparative transcriptomics of *de novo* assembled transcriptomes of congeneric species.

Large scale analysis of transcriptomes in species lacking a sequenced genome requires a reference-free reconstruction of transcript sequences from short NGS reads into contigs using *de novo* transcriptome assembly. For this purpose, several *de novo* assemblers like ABySS [15], MIRA [16], SOAP [17], Trinity [18], Velvet-Oases [19], CLC cell are currently available. However, these tools have been used with different rates of success, depending upon the applications and strategies that are used. Accurate assembly of short reads is a challenging task, particularly in the absence of a reference genome, since *de novo* assembly is computationally more intensive than syntenic mapping, which makes use of a reference genome [20]. Comprehensive optimization of input parameters, specific for an assembler, needs to be explored and established for obtaining maximum length of contigs [21]. For instance, a balanced use of k-mer size is required for different assemblers, although even after this precaution, the results due to different assemblers differ [22]. Administering multiple k-mer lengths, however, allows capturing of greater number of rare transcripts from NGS datasets, although it often leads to chimeric or even mis-assemblies [20].

*De novo* assembly of transcript sequences from polyploid species poses an additional challenge for the correct reconstruction by distinguishing transcripts belonging to homoeologues and paralogues [22]. The problem gets confounded due to transcript isoforms as well. Despite these limitations, a few examples of *de novo* assembly of wheat transcriptomes are available. These were intended for broad range of applications like identification of grain protein content genes [23], expression analysis of fatty acid desaturase gene in response to H_2_O_2_ during powdery mildew infection [24], to understand polyploidization events [25], SNP discovery [26] and identification of genes responsive for development of starchy endosperm [27]. The success rates in these different studies, however, differed. A comparative study of bread wheat non-normalized transcriptomes using two separate *de novo* assemblers Trinity and Trans-ABySS as well as including multiple k-mers of every odd numbers from 45 to 87, provided insights on characterization and identification of new wheat transcripts [28]. Homoeologue specific *de novo* transcriptome assemblies of hexaploid wheat were constructed using a specifically designed bioinformatics pipeline [29]. A more advanced analysis pipeline was used for *de novo* assembly of transcripts of *Triticum urartu*, the AA genome progenitor of wheat and *Triticum turgidum*, the tetraploid pasta wheat with AABB genome [7]. The information was used to generate homoeologue specific sub-assemblies that had been subjected to annotate open reading frames, develop gene models and discerning homoeologue specific SNPs.

The three types of rusts [stem (black), stripe (yellow) and leaf (brown)] are the most severe diseases of wheat causing extensive loss in production worldwide [30]. Leaf rust is the most widespread among the different rust diseases of wheat that brings about 10% yield loss annually. The biotrophic basidiomycete fungus *Puccinia triticina* Eriks., responsible for leaf rust, prefer similar environmental conditions for growth and infection that are also favorable for wheat cultivation, thus complicating control of the disease [30]. About 60 different leaf rust resistant genes had been identified from diverse wild relatives of wheat, and many of them were introgressed and pyramided in wheat cultivars to incorporate genetic resistance. But the rapid evolution of the pathogen along with its proficient spore dispersal mechanism frequently broke down resistance, demanding more in-depth study on this host-pathogen interaction.

The present study was undertaken to identify and interrelate differentially expressed genes, resistant (*R*) genes and the genes encoding transcription factors from *de novo* transcriptome assemblies reconstructed from pathogen infected and mock inoculated resistant and susceptible wheat near-isogenic lines (NILs) harboring seedling resistance gene *Lr28* to better understand the molecular phenomenon underlying wheat-leaf rust interaction. The outcome of this study will help to identify SNPs and design better breeding strategies to counter leaf rust disease.

## Materials and Methods

### Plant materials, growth and pathogen inoculation of plants

Near-isogenic lines of wheat (*Triticum aestivum* L.) cultivar HD2329 with [seedling leaf rust resistant, nest immune, infection type 0–0; (no uredia or dead flecks)] or without [seedling leaf rust susceptible, infection type 3+; (uredia somewhat larger than normal size and may be associated with chlorosis or rarely necrosis)] the seedling leaf rust resistant gene *Lr28* were used in this study [31]. The seeds were sowed in sterile composite soil (peat, sand, soil, 1:1:1). The seedling were grown to single leaf stage (~7 days after germination) in a growth chamber under ideal conditions (22°C, Relative Humidity 80%, 16h light at 300 lux; and 8h of darkness) at National Phytotron Facility, Indian Agricultural Research Institute, New Delhi. The plants were watered once every day with equal amounts of water.

**Fig 1:**
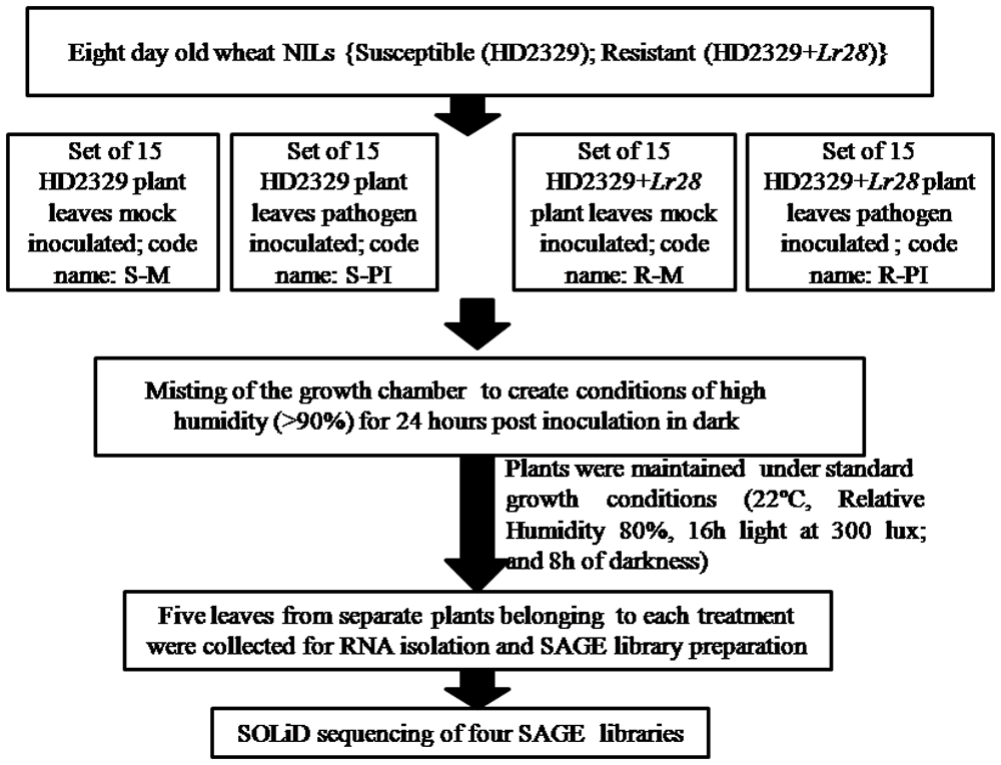
Flowchart illustrating sampling flow.

*Puccinia triticina* pathotype 77–5, the most prevalent and widespread leaf rust pathogen in the Indian subcontinent, was used as the experimental pathogen. The pathogen inoculum was prepared by addition of urediniospores and talcum powder (ratio 1:1) and applied smoothly on leaves of HD2329 + *Lr28* and HD2329. Each treatment combination was applied to 15 plants. The treatment combinations included Talcum powder+Talcum powder in susceptible HD2329 (susceptible negative control; S-M), Talcum powder+*P*. *triticina* urediniospores in susceptible HD2329 (susceptible pathogen infected; S-PI), Talcum powder+Talcum powder in resistant HD2329+*Lr28* (resistant negative control; R-M) and Talcum powder+*P*. *triticina* urediniospores in resistant HD2329+*Lr28* (resistant pathogen infected; R-PI). After inoculation, misting of the growth chamber was performed to create conditions of high humidity (>90%) for 24 hours post inoculation (hpi) in the dark to facilitate infection. Thereafter, the plants were maintained in a standard growth chamber as mentioned earlier [32]. The wheat leaf samples (5 leaves from separate plants of each treatment) were collected at 24 hpi and immediately dipped in liquid nitrogen and kept for RNA isolation (Fig 1). The remaining plants were maintained in the phytotron for another two weeks and checked regularly for appearance of infection symptoms. Infectivity studies and its statistical analysis were performed as mentioned in earlier study [32].

### RNA isolation, SAGE library construction and sequencing

Total RNA was isolated from leaves sampled from five plants for each treatment and were bulked together. The leaf samples were homogenized using liquid nitrogen in a mortar with pestles and RNA was extracted using TRI REAGENT (Molecular Research Center, Inc., USA) as recommended by the manufacturer. The integrity of the isolated RNAs was confirmed by electrophoresis on denaturating formaldehyde-1.2% agarose gel. The RNA was treated with DNase I, amplification grade and RNaseOUT (both component of the SOLiD SAGE kit), at 37°C for 30 min and quantified on a BioPhotometer (Eppendorf AG, Germany).

Four Serial Analysis of Gene Expression (SAGE) libraries were prepared using SOLiD3 System SAGE kit (Applied Biosystems, CA, USA) from the isolated RNA following recommended protocol. The SAGE tags were resolved and purified using two rounds of highly sensitive Agilent 2100 Bioanalyzer for 100 bp fragments where extra bands arising due to over-amplification as well as primer artifacts were removed. The emulsion PCR library prepared were of concentration 500 pM. The amplified emulsion PCR products were subjected to Sequencing by Oligonucleotide Ligation and Detection (SOLiD) on SOLiD3 sequencing platform at Bay Zoltan Foundation of Applied Research, Institute of Plant Genomics, Human Biotechnology and Bioenergy, Zegreb, Hungary.

### Transcriptome assembly

The reads from the four libraries were imported separately to CLC Genomics Workbench 6.5.1 (CLC bio, Aarhus, Denmark; http://www.clcbio.com). Trimming was performed under the following trim settings: (i) removal of low quality sequence (limit = 0.05), (ii) removal of ambiguous nt: maximum of 2 nt allowed, (iii) removal of adapter sequences, (iv) removal of sequences on length: minimum length 20 nt and (v) removal of ribosomal sequences. Since two libraries, S-PI and R-PI, might contain reads originated from *Puccinia* transcripts, all post-trimmed reads were mapped to *Puccinia* transcripts available at The Broad Institute (http://www.broadinstitute.org/annotation/genome/puccinia_group/MultiHome.html). The parameters for mapping were length fraction 0.5, similarity 0.8, mismatch cost 2, insertion cost 3, deletion cost 3 and color error cost 3. The reads that did not match to *Puccinia* transcripts were considered for *de novo* assembly.

**Fig 2:**
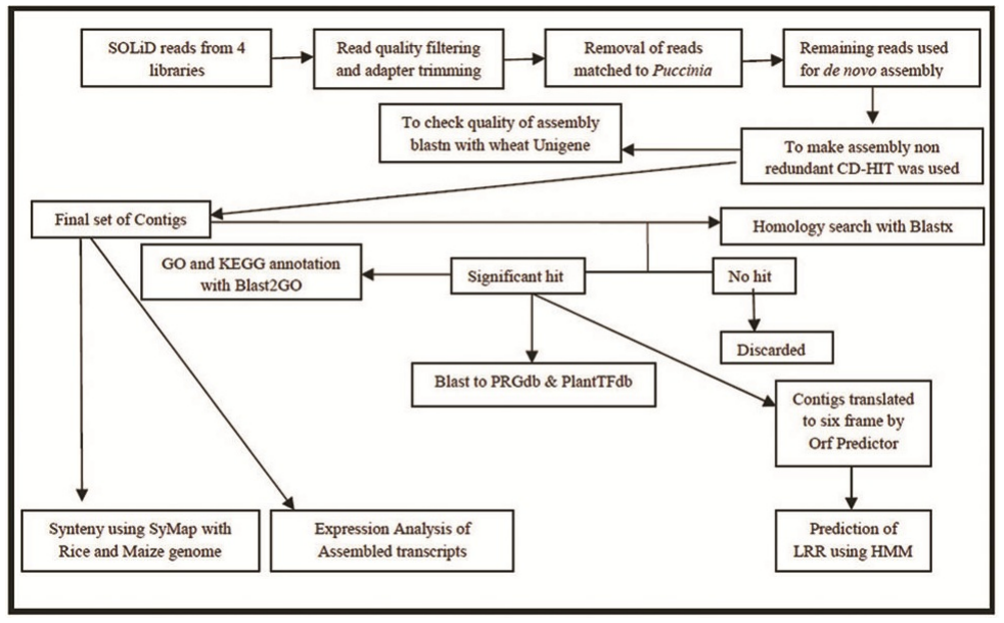
Strategy employed for *de novo* assembly, annotation and expression studies (SOLiD: Sequencing by Oligonucleotide Ligation and Detection; CD-HIT: Cluster Database at High Identity with Tolerance; GO: Gene Ontology; KEGG: Kyoto Encyclopedia of Genes and Genomes; PRGdb: Plant Resistant Gene Database; PlantTFdb: Plant Transcription Factor Database; LRR: Leucine-Rich Repeats; SyMap: Synteny Mapping and Analysis Program; HMM: Hidden Markov model).

Individual assemblies of the four libraries was carried out on a server with 64 GB RAM. CLC Genomics Workbench 6.5.1 was preferred over the other *de novo* assemblers because of its performance in using the parameters that were required for assembly. Assemblies at 15, 17, 19, 21, 23 and 25 k-mers were generated separately for all four libraries. CLC Genomics Workbench *de novo* assembly algorithm, like most currently used *de novo* assembly algorithms, uses de Bruijn graphs [33]. Since 21 and 23 k-mer sizes produced good results; the assembly was also performed for k-mer size 22 for all four libraries. Other parameters included were: bubble size 50 and colorspace alignment, length fractions (match length) of 0.8 and 0.9. To check the quality of the assemblies BLASTN was performed at E-value threshold of 10^−5^ against wheat UniGenes available at NCBI (ftp://ftp.ncbi.nih.gov/repository/UniGene/). Unigene is a transcriptome database available at NCBI. A pipeline was developed for *de novo* assembly of transcripts and to study the assembled contigs (Fig 2). The redundancy was eliminated by choosing only one representative of that sequence using Cluster Database at High Identity with Tolerance (CD-HIT) software [34]. Redundant sequences were removed using CD-HIT-EST run with 95% similarity and subsequently CD-HIT to make clusters of only non-redundant (nr) sequences with similarity of 90%. Assemblies of k-mer 22 were selected for further analysis on the basis of blast hits. The total filtered transcript contigs having length >200 bp were submitted to TSA (Transcriptome Shotgun Assembly) of NCBI database. The primary accession numbers for S-M, S-PI, R-M and R-PI were GBKH00000000, GBKI00000000, GBKJ00000000 and GBKK00000000 respectively.

### Sequence annotation

The assembled sequences were categorized based on homology dependent approach. Blastx was performed against NCBI nr database of proteins (http://www.ncbi.nlm.nih.gov/BLAST/) using an E-value cut-off of 10^−5^. The matched sequences were used for further downstream analysis and poorly supported blast-hit sequences were removed. The matched sequences were annotated with Gene Ontology (GO) terms using Blast2GO (B2G) [35]. Statistically significant enrichment analyses of GO terms present in the assembled contigs were performed in Cytoscape plugin Biological Network Gene Ontology tool (BiNGO) [36] by considering the best blastx hit to *Oryza sativa* proteins. BiNGO hyper-geometric test, with Bonferroni Family-Wise Error Rate correction, was used to determine over-representation [36]. B2G is a similarity search based GO annotation and functional analysis. Whereas, BiNGO is an open-source Java tool that along with Cytoscape [37] helps in visualizing biological networks. It is preferred for determining over-representation of GO terms in sets of genes.

Pathway annotation at Kyoto Encyclopedia of Genes and Genomes (KEGG) was performed using the KEGG Automatic Annotation Server (KAAS) [38] through Single-directional Best Hit (SBH) method. KAAS provides annotation of sequences by BLAST comparisons against the manually curated KEGG database (http://www.genome.jp/kegg/kaas/). The output provides KO (KEGG Orthology) assignments and automatically generated KEGG pathways.

### Identification of transcription factors and resistant genes

To identify transcription factors and decipher their roles during leaf rust-wheat interactions, plant specific transcription factors were downloaded from the Plant Transcription Factor Database (PlantTFdb; http://www.planttfdb.cbi.pku.edu.cn/) [39] version 3.0. Blastx was performed with the *de novo* assembled contigs using an E-value threshold of 10^−5^. Only the top hit for each sequence was considered. Similarly, the *de novo* contigs were also subjected to blastx to Plant Resistant Gene Database (PRGdb; http://www.prgdb.crg.eu/wiki/Main_Page) [40]. PRGdb contains more than 16,000 known and putative *R*-genes belonging to 192 plant species challenged with 115 different pathogens and linked with useful biological information. Also, a Hidden Markov Model (HMM) approach was implemented to search for the profile of Leucine-Rich Repeat (LRR) domains obtained from Pfam [41] to scan the contig sets by the hmmscan program of the HMMER suite [42]. The LRR clan in Pfam consists of 11 families [(DUF285 (PF03382), FNIP (PF05725), LRR_1 (PF00560), LRR_2 (PF07723), LRR_3 (PF07725), LRR_4 (PF12799), LRR_5 (PF13306), LRR_6 (PF13516), LRR_7 (PF13504), LRR_8 (PF13855) and LRR_9 (PF14580)]. Sequences belonging to each family and also NB-ARC sequences were downloaded and the assembled contigs were *in silico* translated to six-frame by OrfPredictor [43]. A stringent domain matching E-value of 0.1 was used to select LRR homologues.

### Analysis of alterations in gene expression among libraries due to leaf rust infection

In order to identify differentially expressed contigs from mock and pathogen inoculated resistant and susceptible wheat NILs, expression analysis was performed based on the abundance of reads within a particular library. For comparison of S-M vs. S-PI, R-M vs. R-PI and S-PI vs. R-PI, the contigs of both libraries were combined to generate the reference. High quality reads from individual library were mapped to the combined contigs to obtain total mapped reads. Analysis of gene expression between the above mentioned pair of libraries were assessed using Reads Per Kilobase of transcript per Million mapped reads (RPKM) where read counts of a particular contig explains its expression. RPKM, allows measuring even sparsely expressed transcripts taking read count as fundamental. The contigs were considered to be differentially expressed when the average fold change was ≤ 2 folds; the other criteria was the false discovery rate (FDR) p-value correction <0.05 and difference in absolute value >10 [44]. All differentially expressed contigs were annotated by B2G and Web Gene Ontology Annotation Plot (WEGO) [45]. WEGO was used for plotting histograms for convenience of visualizing and comparing the results of GO annotation.

All post-trimmed reads were mapped to *de novo* assembled contigs using the minimum read length fraction set at 0.9, minimum similarity set at 0.95, and up to 10 non-specific matches were allowed. RPKM was selected as expression value. Uniquely mapped reads were assigned to each contig, allowing a maximum of two mismatches. Statistical difference in expression level was calculated using Kal’s test [46] at CLC Genomics Workbench.

### Synteny analysis

The assembled contig sequences generated from the four libraries were amalgamated into a single file consisting of only nr contig sequences. The genome of rice pseudomolecules build 7.0 (ftp://ftp.plantbiology.msu.edu/pub/data/Eukaryotic_Projects/o_sativa/annotation_dbs/pseudomolecules/version_7.0/) and maize (ftp://ftp.genome.arizona.edu) was downloaded. Synteny Mapping and Analysis Program v4.0 (SyMap) [47] was used to generate the syntenic relationship with rice and maize. SyMap uses MUMmer [48] program for alignment purpose and passes through four phases (alignment, anchor clustering, anchor filtering and synteny block detection) to compute the synteny blocks.

### Quantitative real-time PCR (qPCR)

Wheat near-isogenic plant types HD2329 with *Lr*28 [resistant (Nest Immune infection typr 0–0;)] and HD2329 lacking *Lr28* (susceptible; infection type 3+) were used for qPCR experiment. Complementary DNA synthesis, as well as qPCR were performed following an earlier study [32]. Sequences for four selected LRR genes were entered in the Universal Probe Library (UPL) assay design centre (www.universalprobelibrary.com) to design long Locked Nucleic Acid (LNA) based short hydrolysis probes as well as forward and reverse primers (S1 Table). The designed probes and primers were synthesized by Roche Diagnostics GmbH, Mannheim, Germany. The qRT-PCR of the selected LRR genes was performed for the samples collected at different time-points same as [32]. The qRT-PCR experiment were performed on a 7,500 Real Time PCR system (Applied Biosystems, USA) in 14 μl reaction volume using 1X FastStart Probe Master (Roche Diagnostics GmbH, Germany) and 200 ng of cDNA. The probe and primer concentration used were 100 nmol and 600 nmol respectively and GAPDH was taken as reference gene. The 96-well optical reaction plates (Thermo Fischer) were incubated at 50°C for 2 min, then at 95°C for 10 min followed by 45 cycles of 95°C for 15 sec and 58°C for 1 min. Three technical replicates of cDNAs prepared from three different plants of each treatment were used for the experiment. Instrument operation, data acquisition and processing were performed employing the Sequence Detection System v 1.2.2 software (Applied Biosystems, USA). Fluorescence signals were collected at each polymerization step and a threshold constant CT value was calculated from the amplification curve by selecting the optimal ΔRn in the exponential region of the amplification plot [49]. Gene expression levels were computed relative to the expression of the reference gene GAPDH at same time points using the 2^-ΔΔCT^ method [50]. Student’s t-test was used to compare data from infected and mock-inoculated materials.

## Results

### SOLiD sequencing of SAGE data

Four SAGE libraries were prepared from wheat plants treated as follows: (i) HD2329 mock inoculated (S-M), (ii) HD2329 pathogen inoculated (S-PI; compatible interaction), (iii) HD2329+*Lr28* mock inoculated (R-M) and (iv) HD2329+*Lr28* pathogen inoculated (R-PI; incompatible interaction). Sequencing of the four SOLiD-SAGE libraries generated a total of 165,767,777 reads, each 35 nucleotides (nts) long with a total of 8 GB data in *.csfasta format (Table 1). The reads were processed following the designed pipeline shown in Fig 2. Trimming resulted in 38,180,500 bases with average high quality scores of 28.95 nts in length. To remove *Puccinia* transcripts that might be present in the reads as contaminants, we mapped the trimmed sequences to the *Puccinia* transcripts. A total of 7,297,731 reads matched to *Puccinia* transcripts, pathogen infected (S-PI and R-PI) samples had comparatively higher similarities (Table 1). In compatible interaction 2,70,1621 reads matched to *Puccinia* transcripts whereas 1,232,943 reads matched to *Puccinia* transcripts in incompatible interaction. The sequencing depth for S-M library was 16˟ and for the remaining three libraries were 13˟.

**Table 1:** Summary of SOLiD SAGE data and mapping to *Puccinia* transcripts.

Library name	No. of reads obtained	No. of reads after trimming	No. of reads mapped to *Puccinia* transcripts	Percentage of reads mapped *Puccinia*
S-M	48,782,889	12,247,862	2,154,694	17.6
S-PI	37,756,220	12,924,486	2,70,1621	20.9
R-M	40,118,870	6,780,611	1,232,943	18.2
R-PI	39,109,798	6,227,541	1,208,473	19.4
Total	165,767,777	38,180,500	7,297,731	

### *De novo* assembly

**Fig 3:**
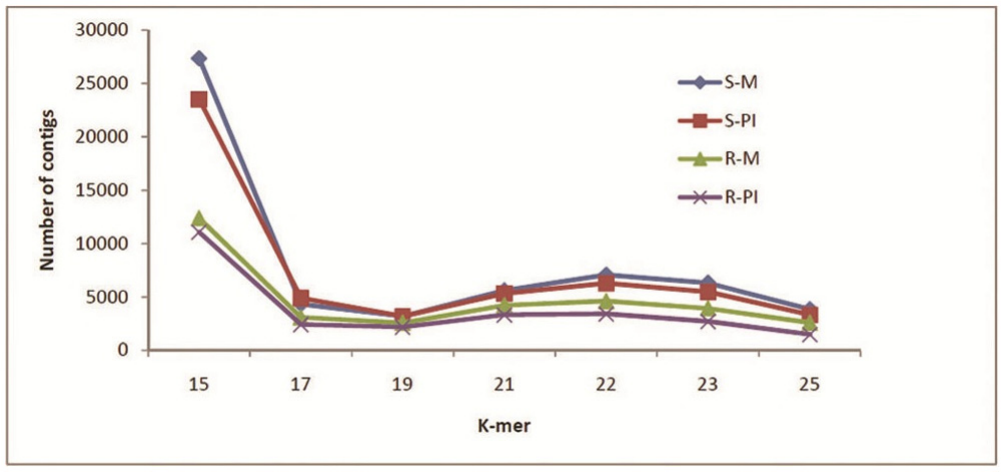
Effect of different k-mer sizes on total number of contigs in the four libraries.

**Fig 4:**
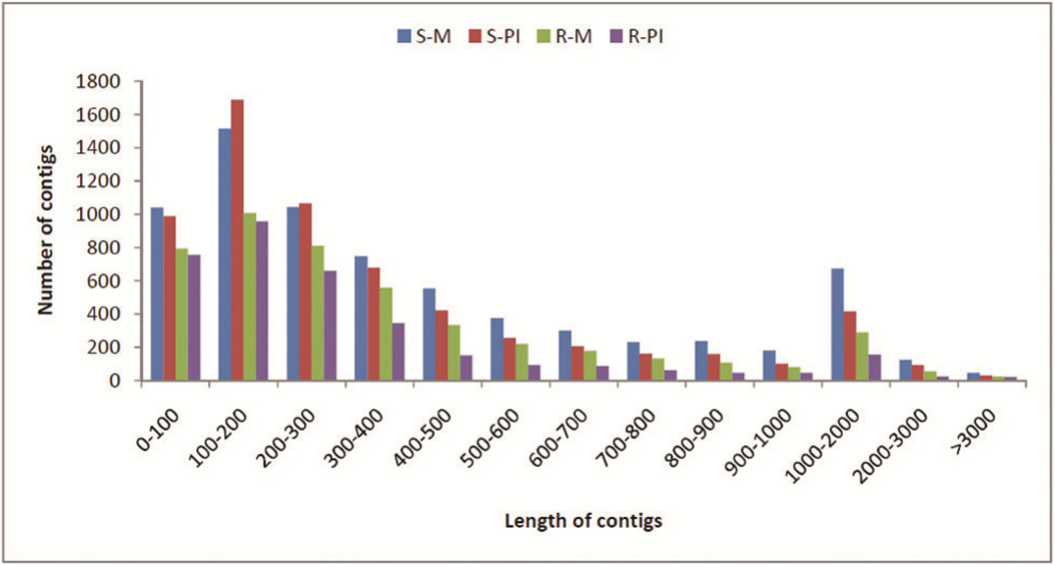
Comparative view of distribution of contigs on the basis of length in four libraries.

An important attribute to consider for *de novo* assembly is the word size (k-mer). Six different k-mers (15, 17, 19, 21, 23 and 25) were initially attempted and since both 21 and 23 provided good results, 22 k-mer was also attempted. The total numbers of contigs obtained were assessed for each k-mer (Fig 3). The number of contigs declined below 15 k-mer and above 23 k-mer sizes but increased at k-mer 21 to 23 (Fig 3). The assembly with k-mer 22 was selected because of its performance on generating the total number of contigs and in order to balance highest and average contig length. Hence, data of each library, was independently assembled at k-mer 22 (Table 2). The length distribution of the contigs in individual library is shown in Fig 4. The reads that passed the stringent quality control, as mentioned under ‘materials and methods’ section, were only considered for *de novo* assembly. The S-M, S-PI, R-M and R-PI libraries had average contig sizes of 484, 410, 409 and 337 nt respectively (Table 2). We found 11.9%, 8.6%, 8% and 5.9% of transcript assembled contigs to be greater than 1000 nt in S-M, S-PI, R-M and R-PI libraries respectively (Table 2).

**Table 2:** Summary of *de novo* assemblies of individual library.

Attributes		Libraries		
	S-M	S-PI	R-M	R-PI
**Base Pairs**				
N50	835	704	651	556
Minimum	33	34	50	54
Maximun	14,236	13,784	13,081	9,335
Average	484	410	409	337
Total length	3,420,409	2,568,342	1,878,558	1,149,571
**Number of contigs**				
Count	7,072	6,270	4,595	3,408
<500	4,904	4,845	3,505	2,871
500–1000	1,322	887	722	334
1000–2000	674	415	289	157
2000–3000	126	93	55	25
>3000	46	30	24	21
CD-HIT-EST	6,949	6,212	4,557	3,396
CD-HIT	6,856	6,145	4,521	3,372

To assess quality of the assembled contigs, the UniGene build 63 of *Triticum aestivum* (as of June 2014) were downloaded and subjected to BLASTN. In S-M library out of 7072 contigs 6595 matched and 477 contigs resulted in no hits. Likewise, in S-PI, R-M and R-PI libraries out of 6270, 4595 and 3408 contigs, 5836, 4316 and 3157 matched whereas 434, 279 and 251 contigs returned no hits respectively. To avoid misassembled contigs only those contigs were selected which provided significant hit in nr protein database at NCBI.

As *de novo* assembly produces numerous redundant contigs which hinder downstream analysis, therefore the redundant contigs were removed by employing CD-HIT-EST first and then CD-HIT. The two programs reduced the number of contigs considerably to the final collection of nr contigs only (Table 2).

### Functional annotation of the *de novo* assembled contigs

The assembled contigs that significantly matched to NCBI nr protein database were assigned GO terms using B2G. A total of 6122 contigs with significant hits were obtained out of which 2409, 1656, 1432 and 625 contigs corresponded to S-M, S-PI, R-M and R-PI respectively. Contigs that did not show any match might be due to either their too short sequence length or might represent novel wheat genes that are not yet annotated and characterized. Most of these annotated contigs showed homology with plants and many of the top hits matched to *Aegilops tauschii* whose genome sequence information is available [51]. The GO terms were assigned for categories: molecular functions, biological processes and cellular components (Table 3). The major processes detected in all four libraries, under molecular function were catalytic activity and binding. Protein binding transcription factor activity was unique to only R-PI library. These findings are congruent to earlier studies with wheat [8, 52]. Under biological process, metabolic and cellular processes preponderate in all four libraries. Interestingly, three new processes *i*.*e*. immune system process, cell killing and biological adhesion were exclusively found under biological processes in R-PI library. The cellular component category discerned organelle and cell as the maximum representation in all four libraries. Symplast and cell junction were unique to R-PI library in this category. The results display the provenance of novel GO terms during leaf rust-wheat incompatible interaction under all three categories.

**Table 3:** Summary of Blast2GO.

Gene Ontology terms	S-M	S-PI	R-M	R-PI
**Annotation of biological process at level 2**				
Single organism process	11.1	11.2	11.0	12.1
Signaling	2.2	2.0	1.7	1.6
Response to stimulus	10.9	10.4	10.9	8.3
reproduction	2.7	2.5	2.8	1.6
Multicellular organismal process	4.7	4.2	4.6	3.6
Multi organism process	0.4	0.3	0.3	1.4
Metabolic process	24.1	25.2	25.4	24.3
Localization	7.4	6.8	7.2	6.6
Growth	1.3	0.8	1.0	0.7
Developmental process	4.9	4.3	5.0	3.7
Cellular process	17.1	18.7	17.8	22.7
Cellular component organization or biogenesis	6.0	6.5	5.7	5.4
Biological regulation	7.2	7.0	6.4	7.4
Immune system process	0.0	0.0	0.0	0.5
Cell killing	0.0	0.0	0.0	0.1
Biological adhesion	0.0	0.0	0.0	0.1
**Annotation of molecular function at level 2**				
Transporter activity	5.90	5.70	6.36	6.21
Translation regulator activity	0.05	0.00	0.00	0.00
Structural molecule activity	2.9	3.48	4.94	4.52
Receptor activity	0.42	0.40	0.16	0.19
Nutrient reservoir activity	0.09	0.13	0.24	0.00
Nucleic acid binding transcription factor activity	1.49	1.68	1.17	1.88
Molecular transducer activity	0.61	0.40	0.39	0.38
Enzyme regulator activity	1.17	1.27	1.57	1.70
Electron carrier activity	1.97	3.02	1.96	2.26
Catalytic activity	42.27	40.68	41.24	39.74
Binding	42.50	42.76	41.32	41.62
Antioxidant activity	0.61	0.46	0.63	0.94
Protein binding transcription factor activity	0.00	0.00	0.00	0.56
**Annotation of cellular component at level 2**				
Organelle	35.31	36.03	35.14	33.58
Membrane enclosed lumen	2.09	2.96	2.57	2.69
Membrane	9.33	8.14	8.14	12.33
Macromolecular complex	7.61	8.18	7.44	9.52
Extracellular region	2.18	2.70	2.04	1.59
Cell	43.48	41.99	36.60	38.58
Cell junction	0.00	0.00	0.00	0.85
Symplast	0.00	0.00	0.00	0.85

All data shown are in %

The three categories were further evaluated with blastx to *Oryza sativa* protein sequences. The statistically significant enrichment analyses of GO terms in the contigs were performed in BiNGO. In S-M, under molecular function (Table A in S1 File) catalytic function was found to be comparatively enriched, under biological process, (Table B in S1 File) cellular process was significantly enriched, whereas under cellular component (Table C in S1 File) intracellular part and plastid were over-represented. In S-PI library under molecular process (Table A in S2 File) binding and also stress response related oxidoreductase activity were over-represented than S-M. This implicates increased activity of Reactive Oxygen Species (ROS) scavenging enzymes in pathogen infested susceptible plants. Under biological process (Table B in S2 File) cellular response to stimulus and cellular process showed enrichment. In the cellular components category (Table C in S2 File), cytoplasmic part and plastid were comparatively enriched indicating intense interaction between host tissue and pathogen alongwith enhanced photosynthesis related activity. In R-M, molecular function (Table A in S3 File) catalytic activity is comparatively over-represented whereas in the biological process (Table B in S3 File) catabolic process of porphyrin and tetrapyrrole were enriched. In cellular component (Table C in S3 File), cytoplasmic part is enriched. In R-PI, biological process, metabolic process and cellular process are over-represented whereas, lipid metabolism and lipid biosynthetic processes were newly enriched terms that were absent in R-M (Table A in S4 File).

We were able to identify 255, 219, 218 and 130 pathways in S-M, S-PI, R-M and R-PI respectively by KAAS. Most of the annotated sequences were associated with amino acid, carbohydrate and lipid metabolism. Other notable pathways were mRNA surveillance pathway, plant pathogen interaction, RNA transport etc. Interestingly many of the R-PI contigs were annotated to glycerolipid, glycerophospholipid, sphingolipid and arachidonic acid metabolism as well as fatty acid degradation that were absent in other three libraries. These lipid metabolism associated pathways, may contribute to regulation of responses to pathogen attack by releasing several lipids and lipid metabolites from membranes that function as signal molecules in the activation of plant defense responses [53]. Carbon metabolism was abundant in S-PI and R-PI, indicating requirement and deployment of more energy by the plants to counteract pathogenesis. Similarly, phenylpropanoid biosynthetic pathways were more in S-PI and R-PI; the role of phenylpropanoids as signal molecules in plant-pathogen interaction is also well established [54]. Comparative distribution of KEGG pathways in S-M vs. S-PI and R-M vs. R-PI is shown in Figure B and C in S4 File.

### Identification of transcription factors and resistant genes

**Fig 5:**
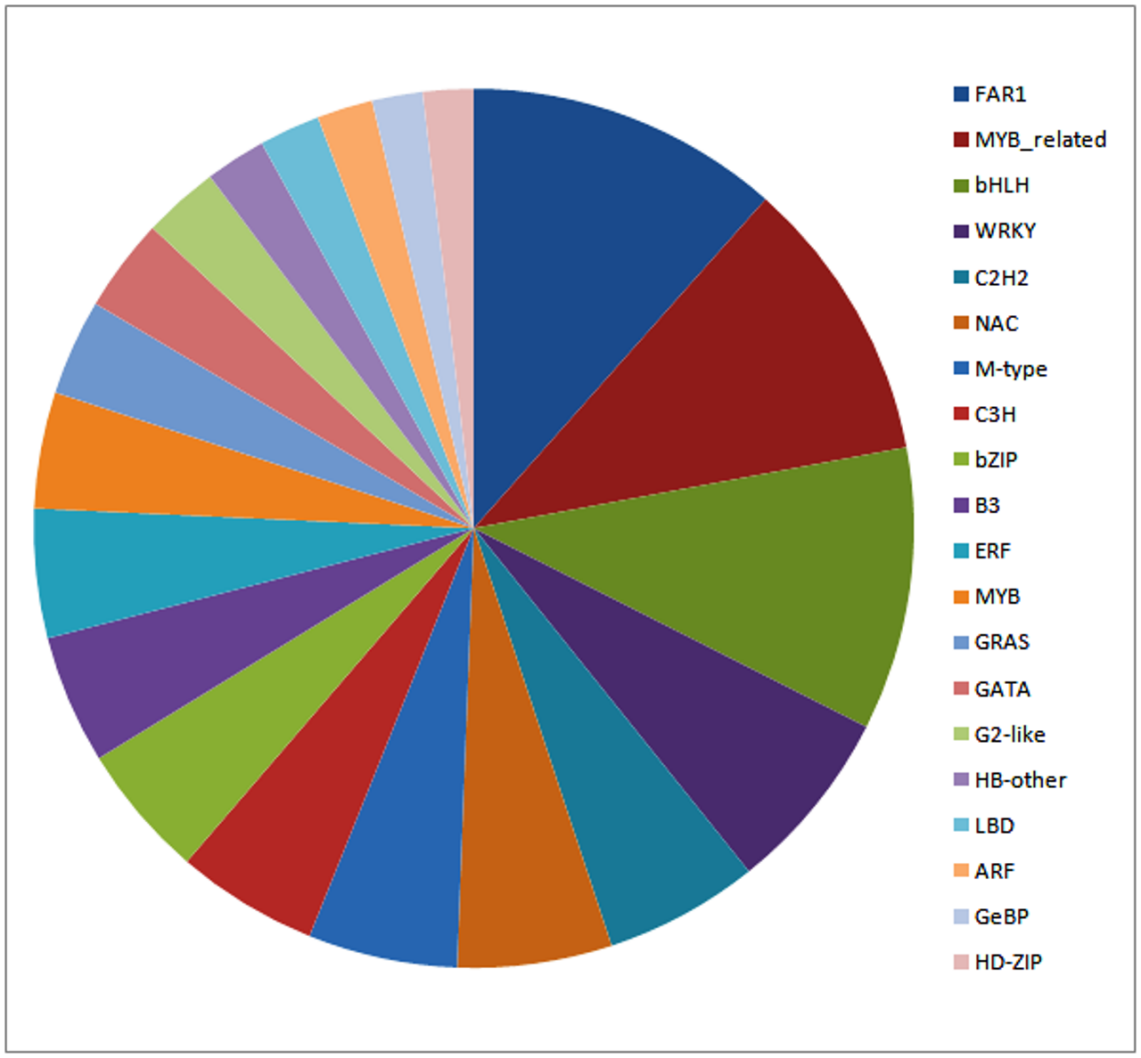
Abundance distribution of transcription factor families in all four libraries.

A total of 1329 *de novo* assembled contigs showed homology with 51 transcription factor families whose sequences are available at PlantTFdb. The twenty most abundant transcription factor families detected were FAR1, MYB_related, bHLH, WRKY, C2H2, NAC, M-type, C3H, bZIP, B3, ERF, MYB, GRAS, GATA, G2-like, HB-other, LBD, ARF, GeBP and HD-ZIP (Fig 5). The roles of MYB, NAC and bZIP transcription factors in plant defense are well known [55, 56]. The upregulation of several WRKY genes in wheat in response to leaf rust has been also documented [57, 58]. The distribution of transcription factor families in all four libraries is provided in S2 Table.

To discern the pathogen induced differentially expressed resistant genes in wheat, contigs from all the four libraries were subjected to blastx in PRGdb. A total of 416 matches with > = 5 hits in contigs were selected and annotated (S3 Table). Protein kinase-like, NB-ARC (nucleotide-binding adaptor shared by APAF-1, R proteins, and CED-4) domain, Leucine-Rich Repeats (LRR), cinnamoyl-CoA reductase, RAG1 (Recombination activating gene1)-Activating protein1 and P-loop containing nucleoside triphosphate hydrolases were some of the prominent resistant genes with similarity to the contigs. FAR1 DNA binding domain which contains a WRKY-like fold and probably a zinc finger DNA-binding domain has been found only in S-PI and R-PI library contigs revealing its counteractive role during pathogen infection. Lr1 disease resistance protein was found exclusively in R-PI library.

Resistance in plants against biotic stress is mainly conferred by nucleotide-binding leucine-rich repeat (NB-LRR) proteins that are under strong diversifying selection [59]. These NB-LRR proteins show high degree of variability in the Leucine-Rich Repeat domains due to pathogen induced stress but the core nucleotide binding site (NB-ARC) has high levels of similarity. A total of 209 hits were obtained with the LRR profile; LRR_8 showing the maximum number (39) of hits and NB-ARC domain with 35 hits. The library specific LRR genes are presented in Table 4. The extracellular type LRR has a consensus sequence of LxxLxLxxNxLxx. The probability of having the similarity outside the consensus amino acid sequence is very less. Different classes of resistant genes detected in the present study include LRR-containing Receptor-Like Kinases (LRR-RLKs), LRR-containing Receptor-Like Proteins (LRR-RLPs), Nucleotide Binding Site LRR proteins (NBS-LRR) and polygalacturonase inhibiting protein. The proteins having the LRR profile in plants have a variety of structures and functions and play key roles against pathogen infection by activating alert signals followed by initiating immunity signals [60].

**Table 4:** Distribution of LRR families and NB-ARC domain in the four libraries.

Domains			Libraries		
	S-M	S-PI	R-M	R-PI	Total
**LRR families**					
LRR_1	10	11	4	2	27
LRR_2	0	1	1	0	2
LRR_4	12	14	5	6	37
LRR_5	1	0	0	0	1
LRR_6	4	7	1	3	15
LRR_7	6	11	1	2	20
LRR_8	12	16	5	6	39
LRR_9	11	11	5	6	33
NB-ARC	14	13	5	3	35
**Total**	70	84	27	28	**209**

All figures represent number of contigs

### Leaf rust infection massively alters gene expression between libraries

Pairwise comparison of contigs between two libraries followed by statistical analysis provided a P value which corresponds to the degree of differential expression of each contig. The contigs were annotated using the B2G software. To identify the wheat genes that are differentially expressed in response to *P*. *triticina* induced infection were compared with WEGO. Therefore, a contig is considered differentially expressed only if it has fulfilled the above mentioned criteria.

#### (a) Comparison during compatible interactions

Comparison between S-M vs. S-PI libraries showed 4260 contigs to have significant differential upregulation of which 2306 contigs in S-M and 1954 contigs in S-PI were upregulated (S5 File). The prominent categories with increase in important transcripts in WEGO were primary metabolism, signaling, membrane, cell wall, binding (DNA and RNA binding) and photosynthesis (S4 Table). Several defense and stress responsive pathogen-inducible, pathogenesis-related and protein kinases were more expressed in S-PI library (S6 File) denoting the susceptible plants strive to arrest progression of the invading pathogen.

To distinguish the complex behaviour of the transcriptome in response to pathogen infection, KEGG database was used to interpret the pathway annotation of the contigs (S7 File). The dominant pathways for upregulated contigs in S-PI include flavonoid, phenylpropanoid, steroid biosynthesis, glycolysis and gluconeogenesis, carbon fixation in photosynthetic organism, oxidative phosphorylation, pentose phosphate pathway, fructose and glycerolipid metabolism, starch and sucrose metabolism These pathways are involved either in the production of protective molecules or provide energy to safeguard the plant against invading pathogen. The downregulated contigs were involved in cutin, suberin and wax biosynthesis and metabolism of porphyrin and chlorophyll indicating decline of cell wall mediated structural protection.

#### (b) Comparison during incompatible interactions

Comparison between R-M vs. R-PI revealed a total of 2463 contigs to have significant differential upregulation of which 1564 contigs in R-M and 899 contigs in R-PI were upregulated (S8 File). The GO terms that showed changes in WEGO are involved in RNA metabolic process and aging with more number of genes in R-PI (S5 Table). List of some important upregulated gene have been provided in S9 File.

The KEGG pathway analysis revealed that carbon fixation in photosynthetic organism, oxidative phosphorylation, glyoxalate and dicarboxylate metabolism, sphingolipid metabolism and glycerolipid metabolism were prominent pathways in upregulated contigs of R-PI whereas starch, sucrose, porphyrin and chlorophyll metabolism were major pathways in downregulated contigs (S10 File).

#### (c) Comparison between pathogen inoculated susceptible and resistant plants

Comparison between S-PI and R-PI revealed 3804 contigs with significant differential upregulation of which 2060 were in S-PI and 1744 contigs in R-PI (S11 File). The prominent GO processes in WEGO were energy, signaling, metabolic process and defense (S6 Table). Major genes upregulated in R-PI include ubiquitin, auxin related and hypersensitive response (HR) genes as well as kinases like MAPKKK with obvious function in plant defence. Major upregulated genes have been provided in S12 File.

KEGG pathway analysis suggested that aminoacyl t-RNA biosynthesis, glycolysis and gluconeogenesis, purine and pyrimidine metabolism, tryptophan, alanine, aspartate and glutamate metabolism, valine, leucine and isoleucine degradation, starch, sucrose and glycerolipid metabolism were among the abundant pathways in upregulated contigs of R-PI. These pathway being mostly involved in amino acid and carbohydrate metabolism, denote their implications in energy production by the plants to counteract pathogenesis. Glutathione metabolism and carbon fixation in photosynthesis were the critical pathways in the downregulated contigs of R-PI. The comparison of KEGG pathways is shown in (S13 File).

### Synteny analysis

**Fig 6:**
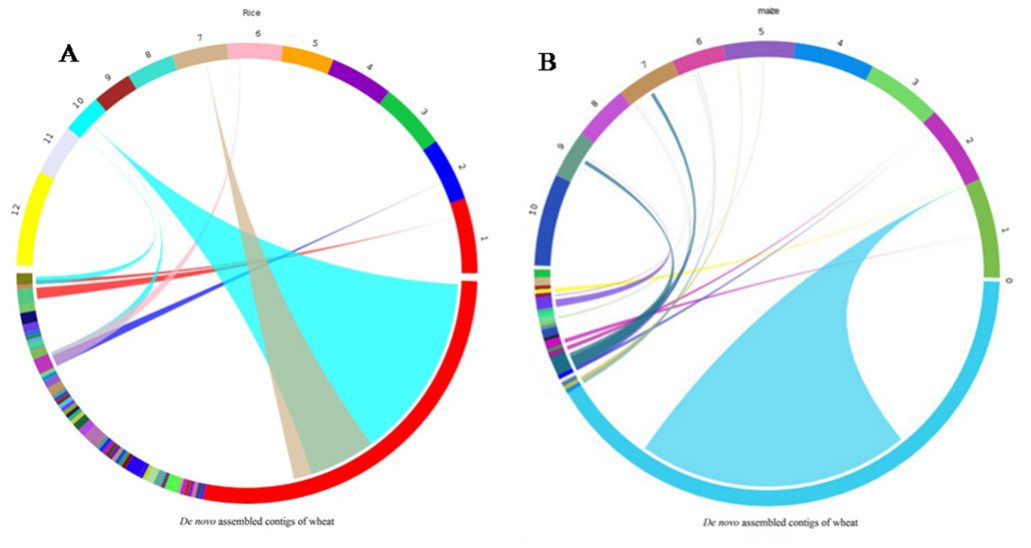
Comparative analyses of *Triticum aestivum* contigs against rice and maize genome. Fig 6A. The SyMAP analysis of *Triticum aestivum* contig blocks against rice genome. The *Triticum aestivum* contigs were first aligned to 12 rice chromosomes based on MUMmer analysis to generate rice wheat synteny contig blocks. Fig 6B. *Triticum aestivum* contig blocks were aligned against 10 maize chromosome based on MuMmer analysis to generate maize wheat synteny contig blocks. Evidence of ancient relationship can be observed when the rice and maize chromosome is aligned with wheat contig blocks as highlighted by colored lines in the two genomes.

The genome of wheat is about 40% and 15.6% larger than that of rice (*Oryza sativa*) and maize (*Zea mays*) respectively. Circle views revealed regions of synteny and colinearity between *de novo* assembled contigs with rice and maize genomes (Fig 6A and 6B). A total anchor of 3078 and 17 blocks were identified with rice with 46% of the total sequence length of wheat *de novo* assembled contigs being covered in the anchor alignment region and 82% of anchors intersected with annotated genes in the rice genome. Comparing with maize genome revealed a total of 5228 anchors and 102 blocks covering 43% of the total wheat *de novo* assembled contig sequence length. Comparative genomics study of the poaceae family that includes rice, maize and wheat might provide several clues for identification of rust resistance and other disease responsive genes.

### Real time qPCR based gene expression

**Fig 7:**
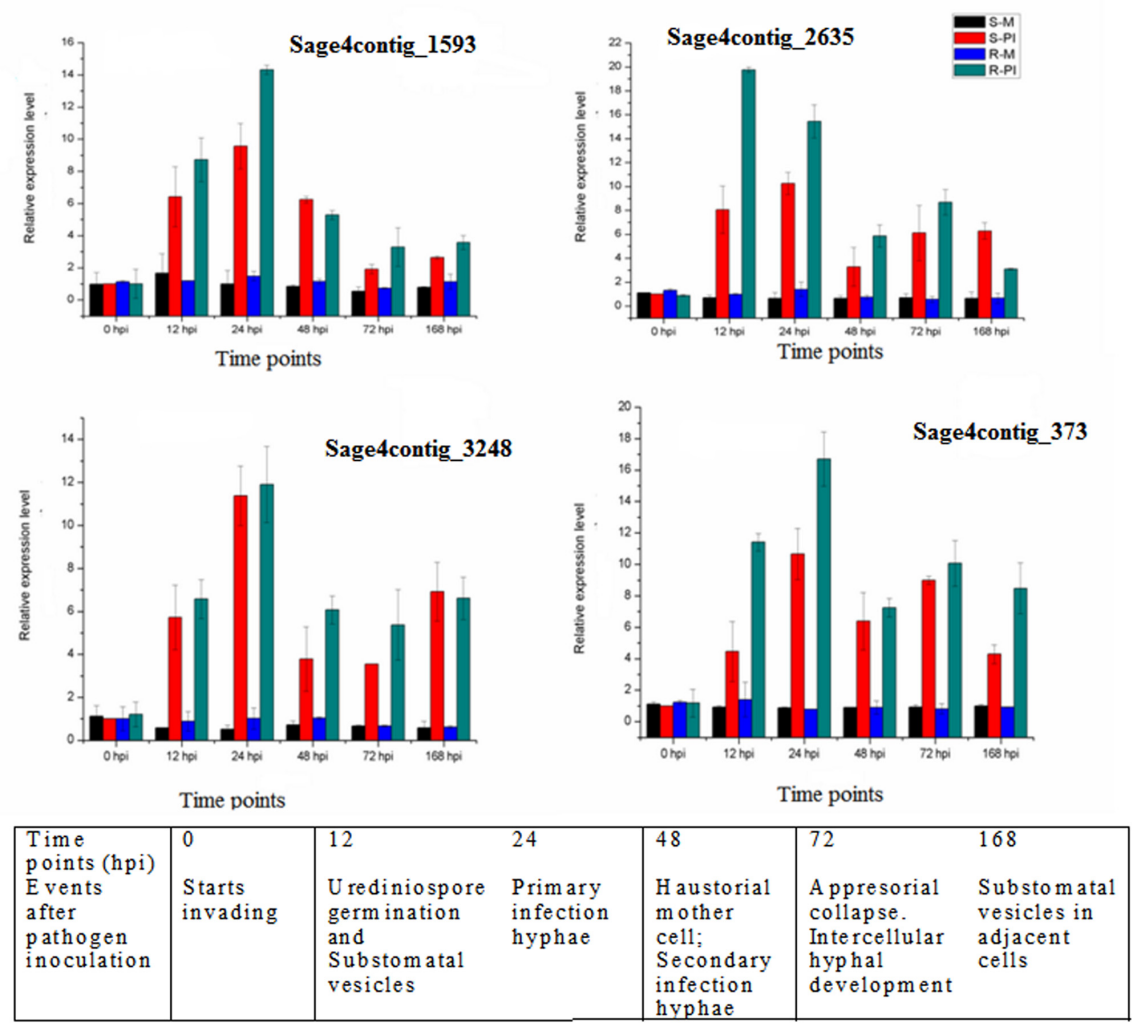
Quantitative real-time PCR (qRT-PCR) analyses of 4 selected LRR-RLK. Leaf tissues were used for both inoculated and mock inoculated plants of resistant and susceptible NILs at 0, 12, 24, 48, 72 and 168 hpi. Mock inoculated susceptible plants at 0 hpi was used as calibrator. Relative gene quantification was calculated by comparative ΔΔCT method. GAPDH expression level was used as internal reference gene and mean ± SD of data from three biological replicates was plotted.

A qPCR assay was used to determine the expression profiles of four LRR-RLK genes positioned on contigs Sage4contig_1593, Sage4contig_2635, Sage4contig_3248 and Sage4contig_373 during different stages of infection by leaf rust pathogen *P*. *triticina* in wheat. The spatial and temporal expression patterns were compared and contrasted in mock- and pathogen-inoculated susceptible and resistant wheat NILs. Development of different infection structures as observed under scanning electron microscopy [61] was correlated with expression of the selected LRR-RLK genes in the present study. The expression increased in all LRR-RLK genes in resistant pathogen inoculated plants, relative to the susceptible pathogen inoculated plants and mock inoculated controls (Fig 7). During incompatible interaction expression was highly induced from 12–24 hpi onwards. This period of maximum expression, 24 hpi, corresponds to the period of proliferation and spread of secondary hyphae to adjacent cells. Collapse of surface mature appresoria initiates the intercellular ramification of secondary infection hyphae at 72 hpi. LRR-RLK genes showed differentially higher expression pattern in inoculated than in uninoculated leaves of both susceptible and resistant NILs. The expression of each of the four genes was higher in the presence of *Lr28* than in its absence. The significance of differences in LRR-RLK gene expression between resistant mock and infected interaction, susceptible mock and infected interaction, and resistant infected and susceptible infected interactions, were evaluated using ΔCt values at p ≤0.01. The t-values obtained between R-M vs. R-PI were 35.107, 113.060, 38.33 and 25.484 for Sage4contig_1593, Sage4contig_2635, Sage4contig_3248 and Sage4contig_373 respectively. The obtained t-values showed significant difference from critical t value (4.604), thus indicating significant difference in expression of the targeted LRR-RLK genes at the point of maximum expression in selected plants.

## Discussion

Response of wheat NILs during compatible and incompatible interactions revealed several genes to be responsible for defense against pathogens. These functionally diverse genes of small effects cumulatively provide quantitative disease resistance (QDR) and are agriculturally important since pathogens rarely evolve new forms which can overcome the combined effects of all these genes [62]. Some of the annotated genes which support the role in regulation of the physiology of the cell (Protein kinase, homologs of LRR, calmodulin, transcription factors) corroborate with earlier articles [63]. The number of genes related to glutathione-*S*-transferase was greater in S-PI than in R-PI suggesting their necessity for the maintenance of lower oxidation state in susceptible host as compared to resistant. Balancing of ROS and antioxidant activity is also an important factor in plant-pathogen interaction [64]. The photosystem center of chloroplast is the origin of ROS and Photosystem II related oxidative burst is required for hypersensitive response (HR). In R-PI, the expression of Pheophorbide a oxygenase (PaO), a homologue of *lethal leaf-spot 1* gene in wheat, is upregulated. PaO, a Rieske-type iron-sulfur cluster containing enzyme is involved in the degradation of pheophorbide a and chlorophyll catabolism [65]. *Lethal leaf-spot 1* serve as a regulator for cell death in plants under various stresses. Rapid increase of H_2_O_2_ after 12 hpi with avirulent *P*. *striiformis* was found to be associated with expression of *lethal leaf-spot 1* gene during incompatible interaction which ultimately spearheaded HR initiation [66]. In R-PI library, reduced expression of aquaporins decreased photosynthesis. Aquaporins also help in checking bacterial multiplication by allowing water in the apoplast as tissue desiccation is an important sign of HR response [67].

Ubiquitin-mediated protein modification also contributes towards defensive role in wheat against *P*. *triticina* [68]. Ubiquitin mediated proteolysis was more in S-PI as compared to S-M whereas, in R-PI, numerous ubiquitin linked proteins like E3 ubiquitin ligase BIG BROTHER-related protein, ubiquitin-like protein 5, ubiquitin carboxyl-terminal hydrolase 5 etc. were overexpressed. In R-PI only, radical induced cell death protein 1 (RCD 1) which protects plant cells from ROS triggered programmes leading to cell death and activation of extra-plastidic antioxidant enzymes was found, denoting the plant’s competence to defy pathogen induced HR [69]. Callose synthase gene which deposits callose in papillae, an active role in penetration resistance, was overexpressed in R-PI library. Callose deposition at sites of fungal penetration provided resistance to the adapted powdery mildew *Golovinomyces cichoracearum* and non adapted powdery mildew *Blumeria graminis* pathogens [70]. A jasmonate signal induced gene, Cystatin-1 precursor, with role in disease resistance was discerned in R-PI. Cystatins help in plant defense by counteracting exogenous proteases secreted by fungi [71]. In grasses, cell wall pectin content is relatively low; the major cell wall hemicellulosic component being heteroxylans comprising mainly of arabinoxylans. Therefore, endo-β-1,4-xylanases are more effective in conquering cell wall rather than pectinases. Therefore, xylanase inhibitors like TAXI-IV and TAXI-III, as found in R-PI, are important genes responsible for plant defense [72].

**Fig 8:**
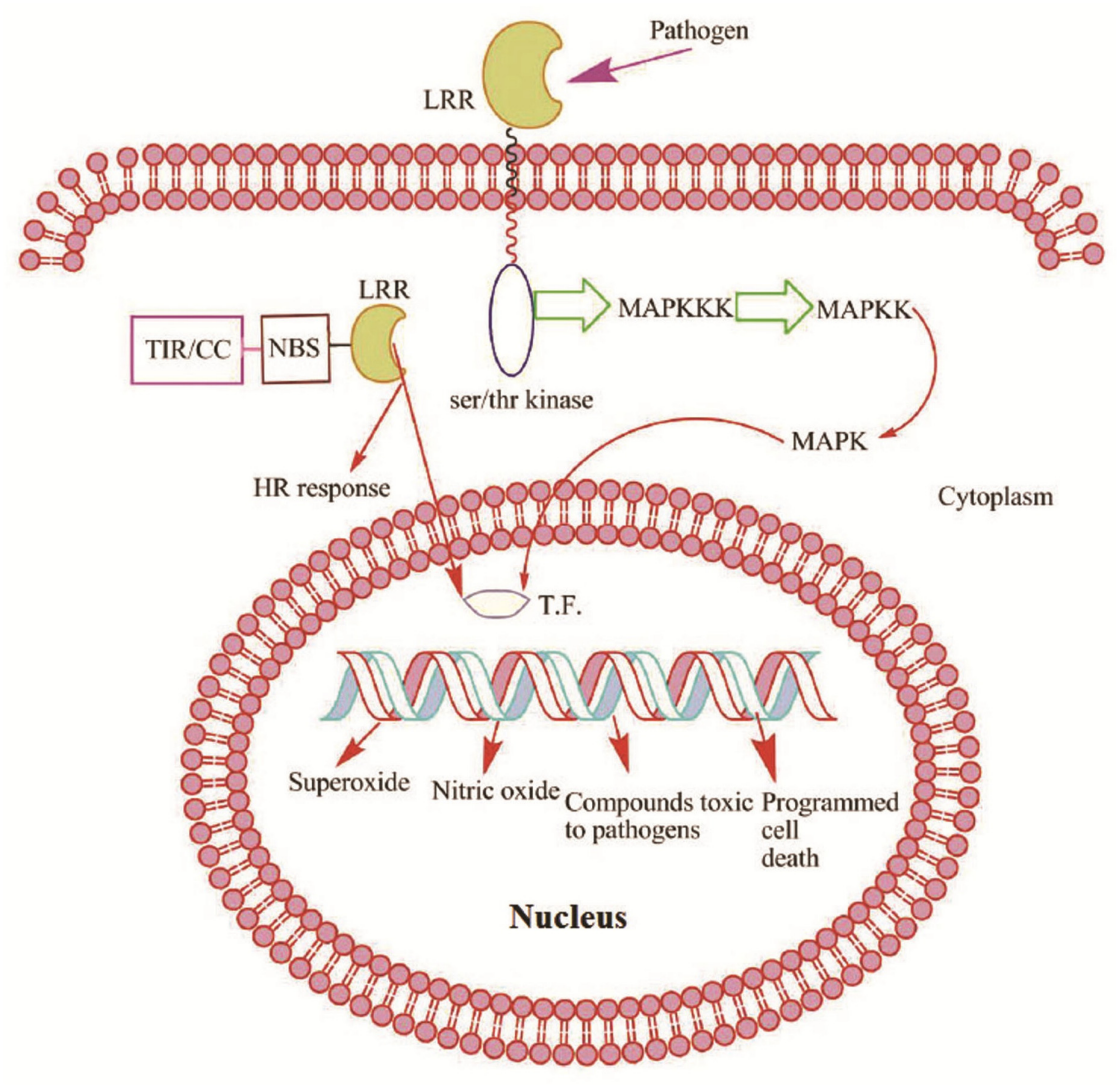
Plants identify pathogen-associated molecular patterns and utilizes extracellular leucine-rich repeat (LRR) receptors for elicitor recognition. Subsequently, serine/threonine kinases activate mitogen-activated protein kinases (MAPKs) for signal relay. NBS-LRR class of *R* genes in plants are divided into two subfamilies: Toll interleukin 1-like receptor (TIR-NBS-LRR) and coiled-coil (CC-NBS-LRR) based on the N-terminus domain carrying the nucleotide binding site (NBS) domain and leucine-rich repeats (LRR) respectively. TIR-NBS-LRRs homologues are rare in monocots. The NBS domain is highly conserved and is utilized to hydrolyze ATP or GTP and the LRR motif is concerned with protein-protein interactions and is responsible for recognition specificity.

The stress response transcriptional coactivator multiprotein bridging factor 1c (MBF1c) is exclusively expressed in R-PI. MBF1c enhances transcripts encoding pathogenesis-related proteins which result in increased tolerance of plants towards pathogens and serve as bridging factor between bZIP transcription factors and TATA box Binding Protein [73]. The increased expression of MBF1c is also associated with several abiotic stresses like drought, heat and salinity. During constitutive expression, MBF1c imparts tolerance to different biotic and abiotic stress. The serine-threonine kinase receptor acts in disease resistance by regulation of development and self-versus non-self-recognition [74]. Protein kinases and phosphatases are essential components of signal transduction pathways that functions through phosphorylation and dephosphorylation of proteins. This leads to a variety of response mechanisms including defense and developmental processes [75]. A model depicting the mode of action of LRR and serine-threonine kinase has been proposed in Fig 8. A Myb_related transcription factor family showed the highest number of upregulated contigs. Role of MAPKs in pathogen defense is well recognized in rice, *Arabidopsis*, tobacco, brassica and tomato [76]. MAPKs belong to Ser/Thr kinases which translate extracellular signals to a wide range of cellular responses. MYB related, MYB, WRKY, NAC, AP-2 and bZIP are the foremost transcription factors that are involved in protein-protein interaction to MAPKs which in turn play the regulatory role in plant defense mechanism [77].

In this study, auxin exhibited antagonistic roles during compatible and incompatible interaction. The expression of auxin regulated proteins like Auxin-induced protein, 1-aminocyclopropane-1-carboxylate oxidase-like, transcription factor ARF are increased in S-PI whereas they are repressed in R-PI. Inoculation of the pathogen *Pseudomonas syringae pv*. *maculicola* with α-Naphthalene acetic acid (NAA) enhanced susceptibility of *Arabidopsis* and accelerated growth of the pathogen [78]. Similarly, accumulation of more Indole 3-Acetic acid (IAA) in susceptible plants as compared to resistant plants was found during infection with different pathogens in rice [79]. The role of auxin in disease development is associated by weakening the plant cell walls, the first barrier against pathogens. Auxin functions in fast elongation of plant tissues by increasing the extensibility of cell wall [80]. The rigidity of cell wall is governed by structural proteins like endo-β-1,4- glucanases, xyloglucan endotransglycosylases and expansin [81]. The expression of endo-β-1,4- glucanases and xyloglucan endotransglycosylases are induced by auxin, and they function during cell wall loosening in association with depolymerization of wall polysaccharides. Initially, pathogens generate IAA to stimulate endo-β-1,4-glucanases and xyloglucan endotransglycosylases to hydrolyze plantcell wall and the released saccharides are used up as nutrition by the pathogen. In this study, in S-PI, the expression of beta-1,3-glucanase precursor was increased in which auxin might have some role. Second, auxins are involved in opening of stomata which may help the pathogen to invade the plant and conquer stoma-conferred plant innate immunity [82]. Third, auxin is antagonistic to salicylic acid-mediated disease resistance pathway [78].

To defend against pathogens, plants depend on innate immunity based on preformed and induced defense responses. Preformed responses are nonspecific and comprise of antimicrobial and structural barriers [83]. The initial defense response includes cytoskeletal reorganization [84]. Cytoskeletal protein binding was conspicuously present in R-PI library and was absent in S-PI library. Energy is essential for the expression of different genes involved in defense pathways [85], therefore, primary metabolism due to pathogen ingression help plants for necessary cellular energy. The upregulated contigs involved in metabolic processes were more in S-PI as compared to S-M but the upregulated contigs in cellular process were more in R-M than in R-PI. Moreover, the upregulated contigs of S-PI are involved in cellular process in more numbers as compared to R-PI. This urges that R-PI is more comfortable in countering pathogen induced stress. Plants under stress upregulate the processes that leads to energy production such as glycolysis, pentose phosphate pathway, tricarboxylic acid cycle, electron transport chain, biosynthesis of ATP and some amino acids such as lysine and methionine whose catabolism leads to the production of energy; whereas glutamic acid, arginine, serine and glycine biosynthesis are upregulated because of their association with photorespiration. The downregulated processes under biotic stress are associated with photosynthesis and starch, lipid and one-carbon metabolism and biosynthesis of amino acids like leucine, isoleucine, and valine [86]. Role of photosynthesis and photorespiration in relation to defense responses are also documented [87]. Photosynthesis is also associated with plant immunity; the infecting pathogen as well as the host plant, both require the products of photosynthesis as carbon sources for energy [88]. Sugar metabolism helps in regulating events associated with defense response such as accumulation of H_2_O_2_ and salicylic acid which elicit HR and helps in expression of defense related genes [89]. Carbohydrate metabolism is also upregulated in both S-PI and R-PI which may be involved in the above mentioned process. Fatty acid metabolism leads to the cascade of defense signaling induced by salicylic acid, jasmonic acid and nitrous oxide. Sphingolipid metabolism helps in salicylic acid-dependent programmed cell death. These processes are dominant in both S-PI and R-PI. When compared R-PI with respect to S-PI, Protein RUPTURED POLLEN GRAIN is uniquely expressed in R-PI [90].

Plant RLKs are transmembrane proteins with putative extracellular domain and intracellular protein kinase domains, which can perceive external signals and switches signal cascades [91]. LRR-RLK flagellin-sensing 2 recognizes one of the most characterized, flagellin (active epitope flg22) which is a component of bacterial flagellum [92]. Similarly, elongation factor Tu (EF-Tu), the most abundant and conserved protein in the bacterial cytoplasm is perceived by LRR-RLK EF-Tu receptor [93]. In Arabidopsis and rice, lysine motif proteins are obligatory for the sensitivity of 10 fungal chitin [94]. In our study the expression of RLK genes were upregulated with maximum upregulation at 24 hpi in resistant HD2329+*Lr28* infected with *P*. *triticina*. This observation is in line with the LRR-RLK gene expression profile based on RNA-Seq and might have role in mediating early events in cotton in response to the wilt fungus *Verticillium dahliae* [95].

In our earlier studies [57,58,32] RNA isolated at different time points after pathogen inoculation from the same plant types that were used for preparation of SAGE libraries, qRT-PCR was performed for target genes encoding chitinase, β-1,3/1,4 glucanase, thaumatin-like protein, peroxidase2, MAPK and several WRKY transcription factor proteins. Resistant plants during incompatible interactions displayed higher levels of expression of target genes quite early (12–24 hpi) as compared to the susceptible plants during compatible interactions where maximum expression was observed at 168 hpi. These differences could be ascribed to the seedling leaf rust resistance gene *Lr28* which prevented leaf rust infection and disease progression in the resistant wheat NILs. Moreover, many genes identified in this study are in concordance with the study carried out in *Arachis diogoi* challenged by late Leaf Spot Pathogen, *Phaeoisariopsis personata* [96]. In another study by our group, cDNA-AFLP (Amplified Fragment Length Polymorphism) was performed during compatible and incompatible leaf rust infection in wheat NILs carrying the recessive *Lr48* gene [97]. As many as 298 transcripts exhibited differential expression, of which 42 transcripts had homology with genes involved in energy production, metabolism, transport, signaling, defense response, plant-pathogen interaction, transcriptional regulation, translation and proteolysis. QRT-PCR also displayed differential induction and attenuation of various transcripts at different stages of leaf rust infection.

## Conclusions

*De novo* assembled wheat transcriptome analysis in the present study revealed differential regulation of numerous genes during *P*. *triticina* induced compatible and incompatible interactions. The products of these induced genes in wheat are mainly proteins with regulatory effect on metabolism, defense and functional activities. Some important GO processes were found in the NILs in response to *P*. *triticina* infection. The knowledge about wheat genes and their interaction is still in its infancy and because of this we received hits with several hypothetical and predicted proteins in the present study. Additionally, a number of genes like *lethal leaf-spot 1*, TIP, RCD1, MBF1c were identified in wheat for the first time that were not known earlier to be associated with defense response against *P*. *triticina*. Focus on these genes will throw light on the expression profile and the part they play in defense and help to identify targets for genetic enhancement of disease resistance.

## Supporting Information

S1 File GO categories enriched significantly in S-M. **(A)** Molecular function **(B)** Biological process **(C)** Cellular Compartment.
(DOC)

S2 File GO categories enriched significantly in S-PI. **(A)** Molecular function **(B)** Biological process **(C)** Cellular component.
(DOC)

S3 File GO terms enriched in R-M. **(A)** Molecular function **(B)** Biological process **(C)** Cellular compartment.
(DOC)

S4 File **(A)** GO terms enriched in R-PI under Molecular function, Biological process and Cellular compartment category. Comparative distribution of different pathways in KEGG database between **(B)** S-M and S-PI and **(C)** R-M and R-PI.
(DOC)

S5 File List of up- and down-regulated transcripts in S-M *vs*. S-PI. (XLS)

S6 File List of some important upregulated genes in S-PI as compared to S-M. (XLSX)

S7 File Comparison of up- and down-regulated contigs of S-PI with respect to S-M in KEGG Pathways. (DOC)

S8 File List of up- and down-regulated transcripts in R-M *vs*. R-PI. (XLS)

S9 File List of some important upregulated genes in R-PI as compared to R-M. (XLSX)

S10 File Comparison of up- and down-regulated contigs of R-PI with respect to R-M in KEGG Pathways. (DOC)

S11 File List of up- and down-regulated transcripts in S-PI *vs*. R-PI. (XLS)

S12 File List of some important upregulated genes in R-PI as compared to S-PI. (XLSX)

S13 File Comparison of up- and down-regulated contigs of R-PI with respect to S-PI in KEGG Pathways. (DOC)

S1 Table Sequences of Primers used in the study. (DOC)

S2 Table Distribution of transcription factor families in the four libraries. (DOC)

S3 Table List of contigs having hits in PRGdb database. (DOC)

S4 Table List of enriched GO terms showing numbers of differentially upregulated contigs of S-M and S-PI. (DOC)

S5 Table List of enriched GO terms showing numbers of differentially upregulated contigs of R-M and R-PI. (DOC)

S6 Table List of enriched GO terms showing numbers of differentially upregulated contigs of S-PI and R-PI. (DOC)
