# Supplementary material for: De Novo Assembled Wheat Transcriptomes Delineate Differentially Expressed Host Genes in Response to Leaf Rust Infection
Source: PLoS One. 2016 Feb 3;11(2):e0148453. doi: 10.1371/journal.pone.0148453 (PMC4739524; doi:10.1371/journal.pone.0148453)
Supplement: S1 File — (A) Molecular function (B) Biological process (C) Cellular Compartment. (DOC) [file pone.0148453.s001.doc]

**S1 File**.

**Table A:** GO categories enriched significantly in S-M under Molecular Function category

| **GO ID** | **Description** | **p-value** | **corr p-value** |
| --- | --- | --- | --- |
| GO:0003824 | catalytic activity | 8.71E-07 | 4.79E-05 |
| GO:0010011 | auxin binding | 1.58E-06 | 8.67E-05 |
| GO:0016667 | oxidoreductase activity, acting on sulfur group of donors | 4.33E-06 | 2.38E-04 |
| GO:0008891 | glycolate oxidase activity | 6.75E-06 | 3.71E-04 |
| GO:0003973 | (S)-2-hydroxy-acid oxidase activity | 6.75E-06 | 3.71E-04 |
| GO:0000822 | inositol hexakisphosphate binding | 2.61E-05 | 1.44E-03 |
| GO:0016899 | oxidoreductase activity, acting on CH-OH group of donors, oxygen as acceptor | 2.61E-05 | 1.44E-03 |
| GO:0043178 | alcohol binding | 4.15E-05 | 2.28E-03 |
| GO:0042562 | hormone binding | 8.00E-05 | 4.40E-03 |
| GO:0034256 | chlorophyll (ide) b reductase activity | 2.44E-04 | 1.34E-02 |
| GO:0016614 | oxidoreductase activity, acting on CH-OH group of donors | 3.67E-04 | 2.02E-02 |
| GO:0005488 | binding | 7.32E-04 | 4.03E-02 |
| GO:0016209 | antioxidant activity | 8.27E-04 | 4.55E-02 |

**Table B:** GO categories enriched significantly in S-M under Biological Process category

| **GO ID** | **Description** | **p-value** | **corr p-value** |
| --- | --- | --- | --- |
| GO:0009987 | cellular process | 1.52E-08 | 3.33E-06 |
| GO:0050896 | response to stimulus | 1.55E-07 | 3.39E-05 |
| GO:0006787 | porphyrin catabolic process | 6.61E-07 | 1.45E-04 |
| GO:0033015 | tetrapyrrole catabolic process | 6.61E-07 | 1.45E-04 |
| GO:0006800 | oxygen and reactive oxygen species metabolic process | 1.19E-06 | 2.60E-04 |
| GO:0009733 | response to auxin stimulus | 1.49E-06 | 3.25E-04 |
| GO:0042221 | response to chemical stimulus | 1.63E-06 | 3.57E-04 |
| GO:0034614 | cellular response to reactive oxygen species | 2.91E-06 | 6.38E-04 |
| GO:0010109 | regulation of photosynthesis | 4.35E-06 | 9.54E-04 |
| GO:0042744 | hydrogen peroxide catabolic process | 4.35E-06 | 9.54E-04 |
| GO:0009853 | photorespiration | 5.15E-06 | 1.13E-03 |
| GO:0006950 | response to stress | 6.88E-06 | 1.51E-03 |
| GO:0000302 | response to reactive oxygen species | 1.15E-05 | 2.52E-03 |
| GO:0044237 | cellular metabolic process | 1.49E-05 | 3.27E-03 |
| GO:0070887 | cellular response to chemical stimulus | 1.56E-05 | 3.41E-03 |
| GO:0042743 | hydrogen peroxide metabolic process | 1.98E-05 | 4.33E-03 |
| GO:0034599 | cellular response to oxidative stress | 3.25E-05 | 7.11E-03 |
| GO:0051716 | cellular response to stimulus | 4.30E-05 | 9.42E-03 |
| GO:0070301 | cellular response to hydrogen peroxide | 4.87E-05 | 1.07E-02 |
| GO:0009628 | response to abiotic stimulus | 6.29E-05 | 1.38E-02 |
| GO:0009734 | auxin mediated signaling pathway | 6.50E-05 | 1.42E-02 |
| GO:0009725 | response to hormone stimulus | 6.50E-05 | 1.42E-02 |
| GO:0008152 | metabolic process | 6.68E-05 | 1.46E-02 |
| GO:0071365 | cellular response to auxin stimulus | 7.40E-05 | 1.62E-02 |
| GO:0009266 | response to temprature stimulus | 7.59E-05 | 1.66E-02 |
| GO:0042542 | response to hydrogen peroxide | 1.20E-04 | 2.62E-02 |
| GO:0009719 | response to endogenous stimulus | 1.23E-04 | 2.70E-02 |
| GO:0009755 | hormone-mediated signaling pathway | 1.32E-04 | 2.89E-02 |
| GO:0019048 | virus-host interaction | 1.32E-04 | 2.89E-02 |
| GO:0010035 | response to inorganic substance | 2.11E-04 | 4.62E-02 |

**Table C:** GO categories enriched significantly in S-M under Cellular Component category

| **GO ID** | **Description** | **p-value** | **corr p-value** |
| --- | --- | --- | --- |
| GO:0044424 | intracellular part | 9.37E-14 | 3.56E-12 |
| GO:0005622 | intracellular | 1.84E-13 | 6.98E-12 |
| GO:0044464 | cell part | 3.49E-13 | 1.33E-11 |
| GO:0005623 | cell | 3.52E-13 | 1.34E-11 |
| GO:0009536 | plastid | 1.29E-12 | 4.90E-11 |
| GO:0005737 | cytoplasm | 1.86E-11 | 7.05E-10 |
| GO:0043231 | intracellular membrane-bounded organelle | 1.41E-10 | 5.34E-09 |
| GO:0043227 | membrane-bounded organelle | 1.43E-10 | 5.44E-09 |
| GO:0044444 | cytoplasmic part | 8.88E-10 | 3.38E-08 |
| GO:0043229 | intracellular organelle | 1.03E-09 | 3.90E-08 |
| GO:0043226 | organelle | 1.05E-09 | 3.99E-08 |
| GO:0019005 | SCF ubiquitin ligase complex | 3.19E-05 | 1.21E-03 |
| GO:0031461 | cullin-RING ubiquitin ligase complex | 1.60E-04 | 6.07E-03 |
| GO:0009507 | chloroplast | 2.57E-04 | 9.78E-03 |
| GO:0000151 | ubiquitin ligase complex | 4.10E-04 | 1.56E-02 |
| GO:0005777 | peroxisome | 6.86E-04 | 2.61E-02 |
| GO:0042579 | microbody | 6.86E-04 | 2.61E-02 |
