# Supplementary material for: De Novo Assembled Wheat Transcriptomes Delineate Differentially Expressed Host Genes in Response to Leaf Rust Infection
Source: PLoS One. 2016 Feb 3;11(2):e0148453. doi: 10.1371/journal.pone.0148453 (PMC4739524; doi:10.1371/journal.pone.0148453)
Supplement: S2 File — (A) Molecular function (B) Biological process (C) Cellular component. (DOC) [file pone.0148453.s002.doc]

**S2 file**

**Table A:** GO categories enriched significantly in S-PI under Molecular Function category

| **GO ID** | **Description** | **p-value** | **corr p-value** |
| --- | --- | --- | --- |
| GO:0005488 | binding | 7.32E-08 | 4.03E-06 |
| GO:0016491 | oxidoreductase activity | 2.48E-06 | 2.21E-04 |
| GO:0016671 | oxidoreductase activity, acting on sulfur group of donors, disulphide as acceptors | 6.22E-06 | 5.54E-04 |
| GO:0000822 | inositol hexakisphosphate binding | 1.25E-05 | 1.11E-03 |
| GO:0022891 | substrate specific transmembrane transporter activity | 1.62E-05 | 1.44E-03 |
| GO:0043178 | alcohol binding | 1.99E-05 | 1.77E-03 |
| GO:0042562 | hormone binding | 3.84E-05 | 3.42E-03 |
| GO:0022892 | substrate specific transporter activity | 3.86E-05 | 3.44E-03 |
| GO:0022857 | transmembrane transporter activity | 6.70E-05 | 5.96E-03 |
| GO:0015075 | ion transmembrane transporter activity | 1.32E-04 | 1.18E-02 |
| GO:0005215 | transporter activity | 1.42E-04 | 1.26E-02 |
| GO:0030744 | luteolin O-methyl transferase activity | 1.70E-04 | 1.52E-02 |
| GO:0051119 | sugar transmembrane transporter activity | 1.84E-04 | 1.64E-02 |
| GO:0015291 | secondary active transmembrane transporter activity | 2.10E-04 | 1.87E-02 |
| GO:0016667 | oxidoreductase activity, acting on sulfur group of donors | 2.24E-04 | 1.99E-02 |
| GO:0015144 | carbohydrate transmembrane transporter activity | 2.82E-04 | 2.51E-02 |
| GO:0008509 | anion transmembrane transporter activity | 3.38E-04 | 3.01E-02 |
| GO:0005364 | maltose:hydrogen symporter activity | 3.41E-04 | 3.03E-02 |
| GO:0008515 | sucrose transmembrane transporter activity | 3.41E-04 | 3.03E-02 |
| GO:0015573 | beta-glucoside transmembrane transporter activity | 3.41E-04 | 3.03E-02 |
| GO:0008506 | sucrose: hydrogen symporter activity | 3.41E-04 | 3.03E-02 |
| GO:0042950 | salicin transmembrane transporter activity | 3.41E-04 | 3.03E-02 |
| GO:0016209 | antioxidant activity | 3.99E-04 | 3.56E-02 |
| GO:0015121 | phosphoenolpyruvate: phosphate antiporter activity | 5.11E-04 | 4.55E-02 |

**Table B:** GO categories enriched significantly in S-PI under Biological Process category

| **GO ID** | | **Description** | **p-value** | **corr p-value** |
| --- | --- | --- | --- | --- |
| GO:0051716 | cellular response to stimulus | | 1.60E-08 | 2.18E-06 |
| GO:0050896 | response to stimulus | | 1.25E-07 | 1.69E-05 |
| GO:0006800 | oxygen and reactive oxygen species metabolic process | | 5.26E-07 | 7.15E-05 |
| GO:0009733 | response to auxin stimulus | | 6.58E-07 | 8.95E-05 |
| GO:0009987 | cellular process | | 9.82E-07 | 1.34E-04 |
| GO:0034614 | cellular response to reactive oxygen species | | 1.29E-06 | 1.76E-04 |
| GO:0042744 | hydrogen peroxide catabolic process | | 2.56E-06 | 3.49E-04 |
| GO:0070887 | cellular response to chemical stimulus | | 3.89E-06 | 5.29E-04 |
| GO:0042221 | response to chemical stimulus | | 4.68E-06 | 6.36E-04 |
| GO:0000302 | response to reactive oxygen species | | 5.12E-06 | 6.96E-04 |
| GO:0042743 | hydrogen peroxide metabolic process | | 1.16E-05 | 1.58E-03 |
| GO:0033554 | cellular response to stress | | 1.23E-05 | 1.67E-03 |
| GO:0034599 | cellular response to oxidative stress | | 1.45E-05 | 1.97E-03 |
| GO:0070301 | cellular response to hydrogen peroxide | | 2.87E-05 | 3.90E-03 |
| GO:0009725 | response to hormone stimulus | | 2.91E-05 | 3.95E-03 |
| GO:0009734 | auxin mediated signaling pathway | | 3.83E-05 | 5.21E-03 |
| GO:0071365 | cellular response to auxin stimulus | | 4.36E-05 | 5.94E-03 |
| GO:0009719 | response to endogenous stimulus | | 5.54E-05 | 7.53E-03 |
| GO:0006979 | response to oxidative stress | | 6.88E-05 | 9.35E-03 |
| GO:0042542 | response to hydrogen peroxide | | 7.07E-05 | 9.62E-03 |
| GO:0009755 | hormone-mediated signaling pathway | | 7.79E-05 | 1.06E-02 |
| GO:0010035 | response to inorganic substance | | 9.51E-05 | 1.29E-02 |
| GO:0009846 | pollen germination | | 2.03E-04 | 2.77E-02 |
| GO:0010304 | PS II associated light harvesting complex II catabolic process | | 2.03E-04 | 2.77E-02 |
| GO:0009657 | plastid organization | | 2.03E-04 | 2.77E-02 |
| GO:0006950 | response to stress | | 2.28E-04 | 3.10E-02 |

**Table C:** GO categories enriched significantly in S-PI under Cellular Component category

| **GO ID** | **Description** | **p-value** | **corr p-value** |
| --- | --- | --- | --- |
| GO:0009536 | plastid | 1.06E-20 | 6.03E-19 |
| GO:0044444 | cytoplasmic part | 2.57E-14 | 1.46E-12 |
| GO:0044424 | intracellular part | 9.37E-14 | 5.34E-12 |
| GO:0043231 | intracellular membrane-bounded organelle | 1.28E-13 | 7.32E-12 |
| GO:0043227 | membrane-bounded organelle | 1.31E-13 | 7.48E-12 |
| GO:0005622 | intracellular | 1.84E-13 | 1.05E-11 |
| GO:0044464 | cell part | 3.49E-13 | 1.99E-11 |
| GO:0005623 | cell | 3.52E-13 | 2.00E-11 |
| GO:0005737 | cytoplasm | 5.40E-13 | 3.08E-11 |
| GO:0043229 | intracellular organelle | 1.33E-12 | 7.57E-11 |
| GO:0043226 | organelle | 1.37E-12 | 7.80E-11 |
| GO:0042170 | plastid membrane | 1.50E-06 | 8.58E-05 |
| GO:0009507 | chloroplast | 1.76E-06 | 1.01E-04 |
| GO:0009526 | plastid envelope | 3.55E-06 | 2.02E-04 |
| GO:0019005 | SCF ubiquitin ligase complex | 3.19E-05 | 1.82E-03 |
| GO:0044435 | plastid part | 7.36E-05 | 4.19E-03 |
| GO:0031461 | cullin-RING ubiqutin ligase complex | 1.60E-04 | 9.11E-03 |
| GO:0000151 | ubiquitin ligase complex | 4.10E-04 | 2.34E-02 |
