# Supplementary material for: De Novo Assembled Wheat Transcriptomes Delineate Differentially Expressed Host Genes in Response to Leaf Rust Infection
Source: PLoS One. 2016 Feb 3;11(2):e0148453. doi: 10.1371/journal.pone.0148453 (PMC4739524; doi:10.1371/journal.pone.0148453)
Supplement: S3 File — (A) Molecular function (B) Biological process (C) Cellular compartment. (DOC) [file pone.0148453.s003.doc]

**S3 file**

**Table A:** GO categories enriched significantly in S-M under Molecular Function category

| **GO ID** | | **Description** | **p-value** | **corr p-value** |
| --- | --- | --- | --- | --- |
| GO:0003824 | catalytic activity | | 2.71E-09 | 4.79E-08 |
| GO:0051119 | sugar transmembrane transporter activity | | 1.16E-08 | 4.10E-07 |
| GO:0015144 | carbohydrate transmembrane transporter activity | | 1.29E-08 | 9.67E-07 |
| GO:0022892 | substrate-specific transporter activity | | 1.66E-06 | 1.24E-04 |
| GO:0022857 | transmembrane transporter activity | | 3.32E-06 | 2.49E-04 |
| GO:0016671 | oxidoreductase activity, acting on sulfur group of donors, disulfide as acceptors | | 7.18E-06 | 5.38E-04 |
| GO:0005215 | transporter activity | | 8.54E-06 | 6.40E-04 |
| GO:0022891 | substrate-specific transmembrane transporter activity | | 2.19E-05 | 1.64E-03 |
| GO:0016667 | oxidoreductase activity, acting on sulfur group of donors | | 2.58E-04 | 1.93E-02 |
| GO:0009670 | triose-phosphate: phosphate antiporter activity | | 3.65E-04 | 2.74E-02 |
| GO:0015121 | phosphoenolpyruvate:phosphate antiporter activity | | 5.48E-04 | 4.11E-02 |
| GO:0008843 | endochitinase activity | | 5.48E-04 | 4.11E-02 |

**Table B:** GO categories enriched significantly in S-M under Biological process category

| **GO ID** | **Description** | **p-value** | **corr p-value** |
| --- | --- | --- | --- |
| GO:0006787 | porphyrin catabolic process | 2.23E-07 | 3.30E-05 |
| GO:0033015 | tetrapyrrole catabolic process | 2.23E-07 | 3.30E-05 |
| GO:0006979 | response to oxidative stress | 3.46E-07 | 5.12E-05 |
| GO:0042744 | hydrogen peroxide catabolic process | 1.47E-06 | 2.18E-04 |
| GO:0006950 | response to stress | 2.55E-06 | 3.77E-04 |
| GO:0050896 | response to stimulus | 2.90E-06 | 4.30E-04 |
| GO:0009628 | response to abiotic stimulus | 6.60E-06 | 9.77E-04 |
| GO:0042743 | hydrogen peroxide metabolic process | 6.68E-06 | 9.89E-04 |
| GO:0009651 | response to salt stress | 1.32E-05 | 1.96E-03 |
| GO:0070301 | cellular response to hydrogen peroxide | 1.65E-05 | 2.44E-03 |
| GO:0009987 | cellular response | 1.78E-05 | 2.64E-03 |
| GO:0042542 | response to hydrogen peroxide | 4.06E-05 | 6.01E-03 |
| GO:0006800 | oxygen and reactive oxygen species metabolic process | 6.66E-05 | 9.85E-03 |
| GO:0034614 | cellular response to reactive oxygen species | 1.21E-04 | 1.79E-02 |
| GO:0006778 | porphyrin metabolic process | 1.40E-04 | 2.07E-02 |
| GO:0033013 | tetrapyrrole metabolic process | 1.40E-04 | 2.07E-02 |
| GO:0006788 | heme oxidation | 1.55E-04 | 2.30E-02 |
| GO:0010304 | PSII associated light-harvesting complex II catabolic process | 1.55E-04 | 2.30E-02 |
| GO:0009657 | plastid organization | 1.55E-04 | 2.30E-02 |
| GO:0009413 | response to flooding | 1.55E-04 | 2.30E-02 |
| GO:0006970 | response to osmotic stress | 1.68E-04 | 2.49E-02 |
| GO:0051187 | cofactor catabolic process | 2.08E-04 | 3.09E-02 |
| GO:0044248 | cellular catabolic process | 2.58E-04 | 3.82E-02 |
| GO:0000302 | response to reactive oxygen species | 3.01E-04 | 4.46E-02 |
| GO:0015996 | chlorophyll catabolic process | 3.11E-04 | 4.60E-02 |

**Table C:** GO categories enriched significantly in S-M under Cellular Component category

| **GO ID** | **Description** | **p-value** | **corr p-value** |
| --- | --- | --- | --- |
| GO:0044464 | cell part | 3.49E-13 | 1.68E-11 |
| GO:0005623 | cell | 3.52E-13 | 1.69E-11 |
| GO:0009536 | plastid | 1.29E-12 | 6.19E-11 |
| GO:0043231 | intracellular membrane-bounded organelle | 4.91E-12 | 2.36E-10 |
| GO:0043227 | membrane-bounded organelle | 5.01E-12 | 2.41E-10 |
| GO:0005737 | cytoplasm | 1.86E-11 | 8.91E-10 |
| GO:0044444 | cytoplasmic part | 3.56E-11 | 1.71E-09 |
| GO:0043229 | intracellular organelle | 4.27E-11 | 2.05E-09 |
| GO:0043226 | organelle | 4.38E-11 | 2.10E-09 |
| GO:0044424 | intracellular part | 1.50E-10 | 7.18E-09 |
| GO:0005622 | intracellular | 2.67E-10 | 1.28E-08 |
| GO:0016021 | integral to membrane | 7.62E-05 | 3.66E-03 |
| GO:0031224 | intrinsic to membrane | 9.93E-05 | 4.76E-03 |
| GO:0005887 | integral to plasma membrane | 1.01E-04 | 4.85E-03 |
| GO:0031226 | intrinsic to plasma membrane | 1.51E-04 | 7.24E-03 |
| GO:0044425 | membrane part | 2.14E-04 | 1.03E-02 |
| GO:0009507 | chloroplast | 2.57E-04 | 1.24E-02 |
| GO:0016020 | membrane | 1.03E-03 | 4.94E-02 |
