# Supplementary material for: De Novo Assembled Wheat Transcriptomes Delineate Differentially Expressed Host Genes in Response to Leaf Rust Infection
Source: PLoS One. 2016 Feb 3;11(2):e0148453. doi: 10.1371/journal.pone.0148453 (PMC4739524; doi:10.1371/journal.pone.0148453)
Supplement: S4 File — (A) GO terms enriched in R-PI under Molecular function, Biological process and Cellular compartment category. Comparative distribution of different pathways in KEGG database between (B) S-M and S-PI and (C) R-M and R-PI. (DOC) [file pone.0148453.s004.doc]

**S4 file**

**Table S4 (A):** GO categories enriched significantly in S-M under Molecular Function, Biological Process and Cellular Component category

| **GO ID** | **Description** | **p-value** | **corr p-value** |
| --- | --- | --- | --- |
| GO:0046523 | S-methyl-5-thioribose-1-phosphate isomerase activity | 6.94E-04 | 9.02E-03 |
| GO:0004557 | alpha-galactosidase activity | 1.10E-03 | 1.42E-02 |
| GO:0015925 | galactosidase activity | 2.41E-03 | 3.13E-02 |
| GO:0016861 | intramolecular oxidoreductase activity | 2.63E-03 | 3.42E-02 |
| GO:0046477 | glyosylceramide catabolic process | 5.98E-04 | 3.71E-02 |
| GO:0046521 | sphingoid catabolic process | 6.22E-04 | 3.86E-02 |
| GO:0046514 | ceramide catabolic process | 6.22E-04 | 3.86E-02 |
| GO:0043102 | amino acid salvage | 6.70E-04 | 4.15E-02 |
| GO:0019509 | L-methionine salvage from methylthioadenosine | 6.70E-04 | 4.15E-02 |
| GO:0071267 | L-methionine salvage | 7.17E-04 | 4.45E-02 |
| GO:0030149 | sphingolipid catabolic process | 7.41E-04 | 4.60E-02 |
| GO:0006677 | glyosylceramide metabolic process | 7.65E-04 | 4.74E-02 |
| GO:0005737 | cytoplasm | 9.56E-07 | 1.53E-05 |
| GO:0044424 | intracellular part | 9.61E-06 | 1.54E-04 |
| GO:0005622 | intracellular | 1.22E-05 | 1.95E-04 |
| GO:0009536 | plastid | 6.05E-05 | 9.68E-04 |
| GO:0044464 | cell part | 7.06E-05 | 1.13E-03 |
| GO:0005623 | cell | 7.07E-05 | 1.13E-03 |
| GO:0009505 | plant-type cell wall | 3.98E-04 | 6.36E-03 |
| GO:0044444 | cytoplasmic part | 5.58E-04 | 8.92E-03 |
| GO:0043231 | intracellular membrane-bounded organelle | 8.49E-04 | 1.36E-02 |
| GO:0043227 | membrane-bounded organelle | 8.54E-04 | 1.37E-02 |
| GO:0043229 | intracellular organelle | 1.56E-03 | 2.50E-02 |
| GO:0043226 | organelle | 1.57E-03 | 2.52E-02 |


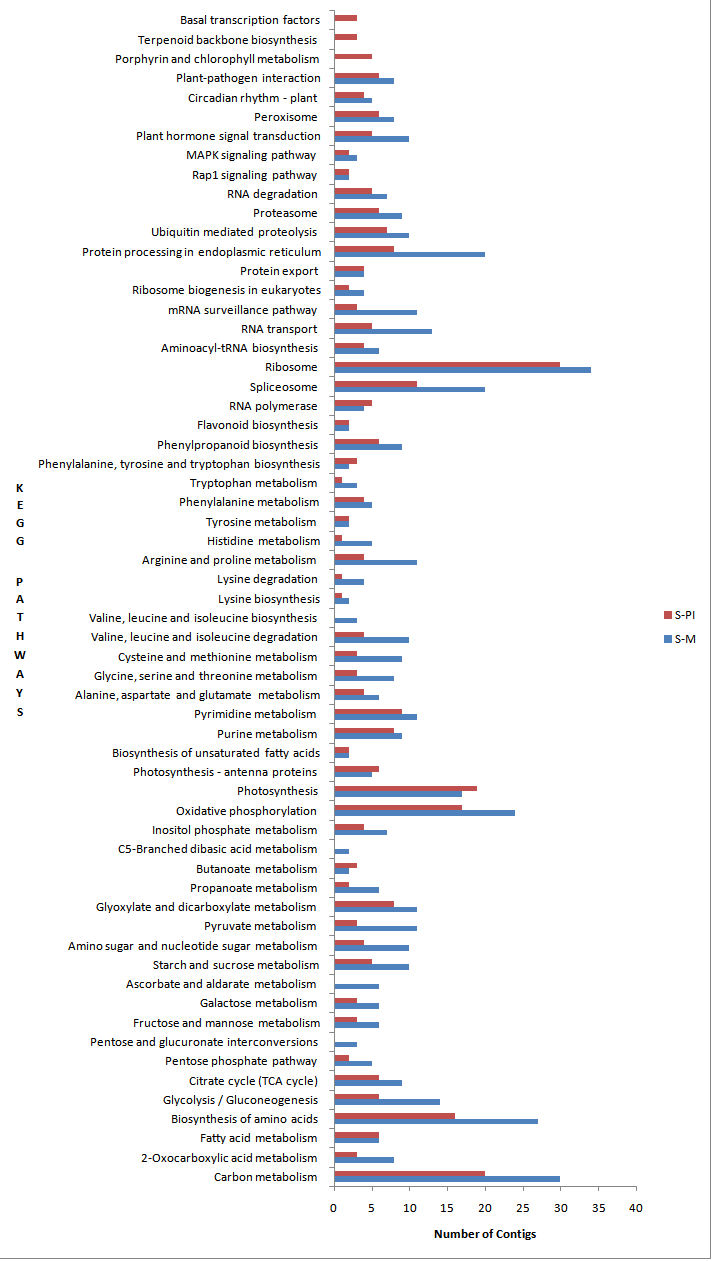


**B**


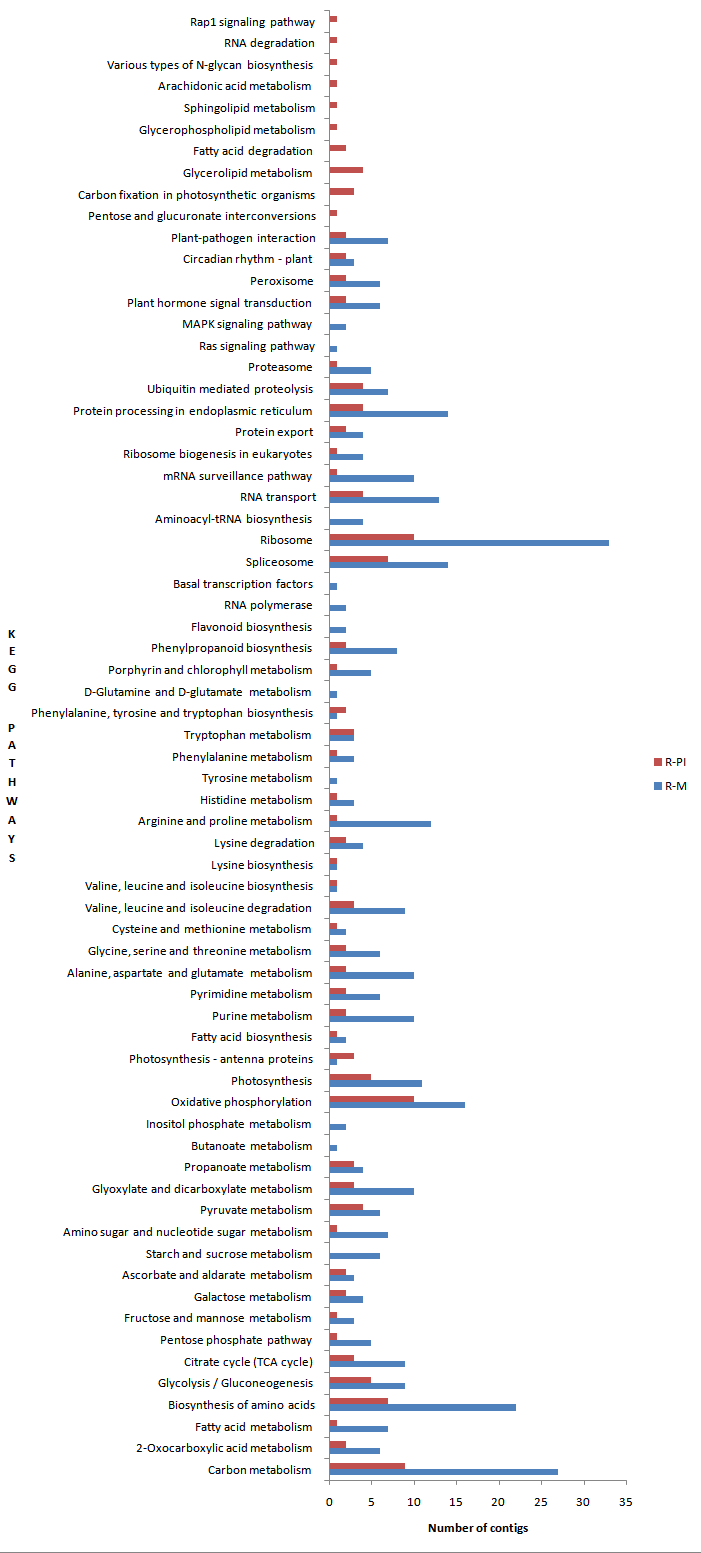


**C**

Comparative distribution of different pathways in KEGG database between (B) S-M and S-PI and (C) R-M and R-PI.
