# Supplementary figures and images for: De Novo Assembled Wheat Transcriptomes Delineate Differentially Expressed Host Genes in Response to Leaf Rust Infection
Source: PLoS One. 2016 Feb 3;11(2):e0148453. doi: 10.1371/journal.pone.0148453 (PMC4739524; doi:10.1371/journal.pone.0148453)

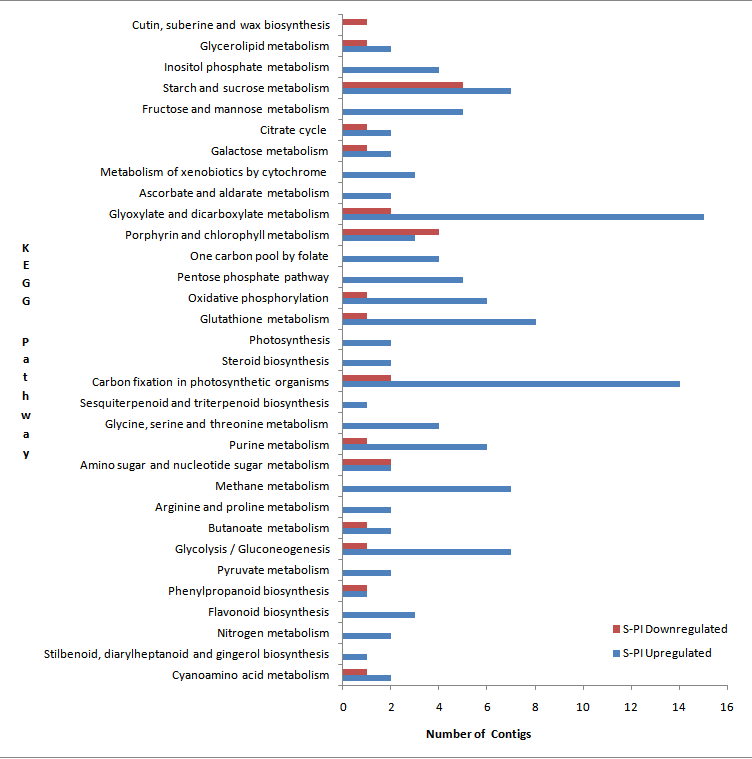


**S7 File**. Comparison of up- and down-regulated contigs of S-PI with respect to S-M in KEGG Pathways

Supplement: S7 File — (DOC) [file pone.0148453.s007.doc]

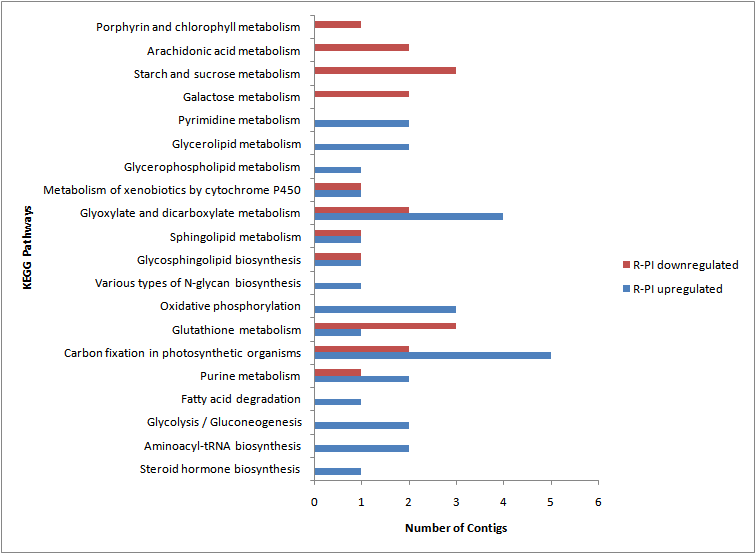


**S10 File**. Comparison of up- and down-regulated contigs of R-PI with respect to R-M in KEGG Pathways

Supplement: S10 File — (DOC) [file pone.0148453.s010.doc]

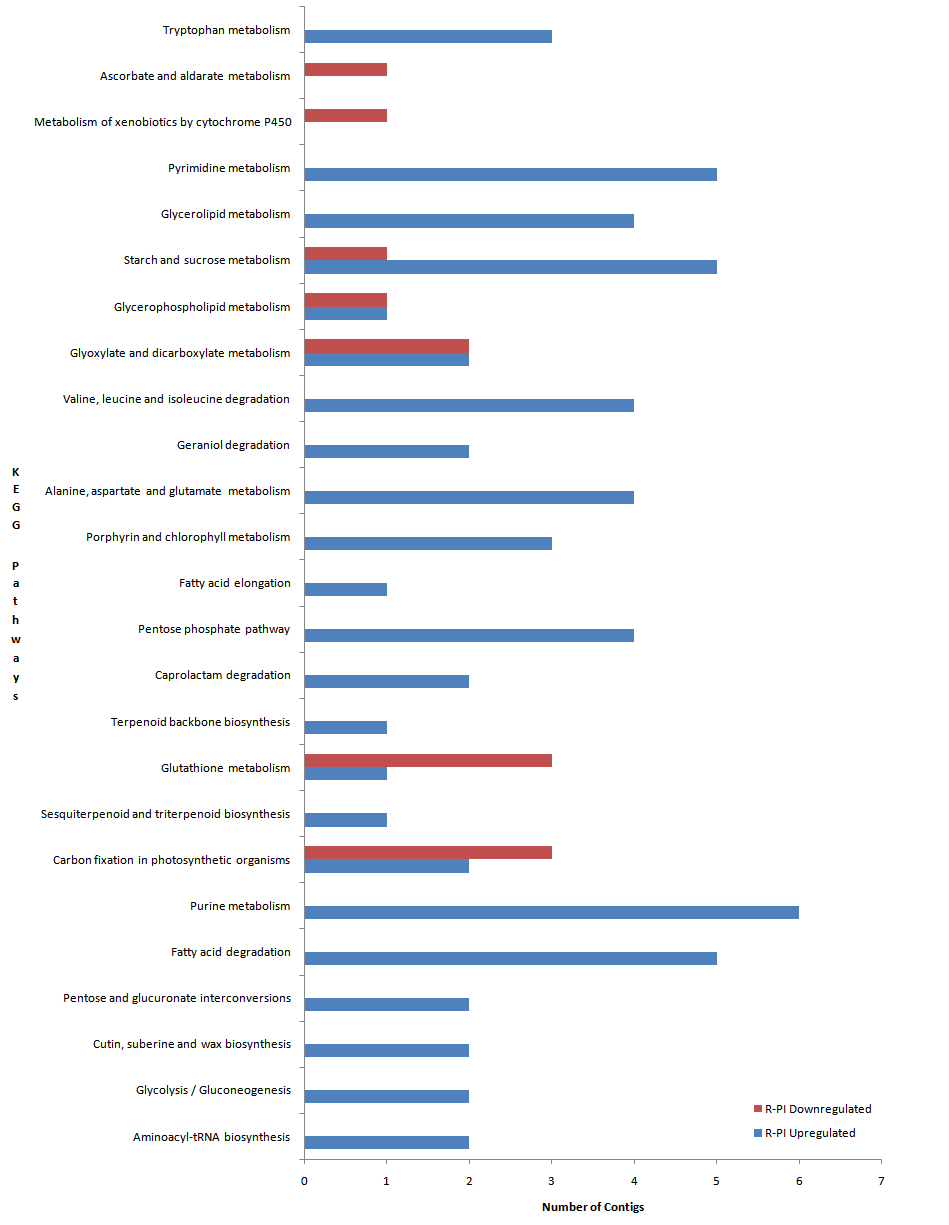


**S13 File**. Comparison of up- and down-regulated contigs of R-PI with respect to S-PI in KEGG Pathways.

Supplement: S13 File — (DOC) [file pone.0148453.s013.doc]
