# Supplementary material for: De Novo Assembled Wheat Transcriptomes Delineate Differentially Expressed Host Genes in Response to Leaf Rust Infection
Source: PLoS One. 2016 Feb 3;11(2):e0148453. doi: 10.1371/journal.pone.0148453 (PMC4739524; doi:10.1371/journal.pone.0148453)
Supplement: S1 Table — (DOC) [file pone.0148453.s014.doc]

**S1 Table: Sequences of the Primers used in the study.**

| **Primer name** | **Sequence (5'-3')** | **UPL Probe#** | **Probe sequence** |
| --- | --- | --- | --- |
| Sage4contig_1593 F | ATTGCCATTGACACATCGAG | 55 | TCCTCTCC |
| Sage4contig_1593 R | GGCGCAATGTACCTAGGATT | 55 | TCCTCTCC |
| Sage4contig_2635 F | TCCTGTAACGGGTTGTCCTC | 87 | CTGCCACC |
| Sage4contig_2635 R | AACATGAGCTCCAGCAGTTG | 87 | CTGCCACC |
| Sage4contig_3248 F | TCGATGAACTGCCGGAATA | 46 | GCAGCCAT |
| Sage4contig_3248 R | TCATGCCTAACGGGAATTTG | 46 | GCAGCCAT |
| Sage4contig_373 F | AGCTGATTGCGCTGTATCTG | 66 | CAGCAGCC |
| Sage4contig_373 R | CCAGATCATCCGGAATTTTG | 66 | CAGCAGCC |
| GAPDH F | AGGAAAAGATGCCTGCATTG | 65 | CTGGAGGA |
| GAPDH R | CTATGTTTGCCGCGACTAGA | 65 | CTGGAGGA |
