# Supplementary material for: De Novo Assembled Wheat Transcriptomes Delineate Differentially Expressed Host Genes in Response to Leaf Rust Infection
Source: PLoS One. 2016 Feb 3;11(2):e0148453. doi: 10.1371/journal.pone.0148453 (PMC4739524; doi:10.1371/journal.pone.0148453)
Supplement: S2 Table — (DOC) [file pone.0148453.s015.doc]

**S2 Table: Distribution of transcription factors families in the four libraries**

| **Family** | **S-M** | **S-PI** | **R-M** | **R-PI** | **Total** |
| --- | --- | --- | --- | --- | --- |
| **FAR1** | 58 | 31 | 19 | 16 | 124 |
| **MYB_related** | 48 | 24 | 29 | 12 | 113 |
| **bHLH** | 45 | 37 | 25 | 5 | 112 |
| **WRKY** | 35 | 21 | 11 | 6 | 73 |
| **C2H2** | 25 | 14 | 15 | 7 | 61 |
| **NAC** | 28 | 14 | 15 | 4 | 61 |
| **M-type** | 18 | 21 | 12 | 8 | 59 |
| **C3H** | 16 | 12 | 16 | 12 | 56 |
| **bZIP** | 24 | 14 | 9 | 6 | 53 |
| **B3** | 16 | 14 | 17 | 4 | 51 |
| **ERF** | 21 | 13 | 13 | 4 | 51 |
| **MYB** | 15 | 16 | 13 | 2 | 46 |
| **GRAS** | 13 | 14 | 9 | 2 | 38 |
| **GATA** | 16 | 7 | 11 | 3 | 37 |
| **G2-like** | 12 | 11 | 5 | 2 | 30 |
| **HB-other** | 12 | 6 | 4 | 2 | 24 |
| **LBD** | 10 | 7 | 3 | 4 | 24 |
| **ARF** | 5 | 7 | 6 | 4 | 22 |
| **GeBP** | 6 | 9 | 3 | 2 | 20 |
| **HD-ZIP** | 5 | 9 | 4 | 2 | 20 |
| **E2F/DP** | 10 | 5 | 2 | 2 | 19 |
| **CAMTA** | 8 | 5 | 3 | 0 | 16 |
| **TCP** | 6 | 5 | 4 | 1 | 16 |
| **DBB** | 7 | 1 | 7 | 0 | 15 |
| **MIKC** | 7 | 2 | 3 | 3 | 15 |
| **YABBY** | 5 | 6 | 3 | 1 | 15 |
| **TALE** | 4 | 6 | 2 | 2 | 14 |
| **CO-like** | 3 | 2 | 4 | 3 | 12 |
| **Dof** | 5 | 4 | 2 | 1 | 12 |
| **HSF** | 7 | 2 | 3 | 0 | 12 |
| **EIL** | 3 | 3 | 4 | 1 | 11 |
| **NF-YB** | 4 | 3 | 2 | 2 | 11 |
| **S1Fa-like** | 3 | 3 | 1 | 2 | 9 |
| **NF-YA** | 4 | 2 | 0 | 2 | 8 |
| **SBP** | 4 | 2 | 2 | 0 | 8 |
| **Trihelix** | 3 | 4 | 1 | 0 | 8 |
| **Nin-like** | 2 | 2 | 2 | 1 | 7 |
| **AP2** | 1 | 3 | 1 | 1 | 6 |
| **ARR-B** | 4 | 2 | 0 | 0 | 6 |
| **WOX** | 2 | 2 | 1 | 1 | 6 |
| **BBR-BPC** | 1 | 3 | 1 | 0 | 5 |
| **NF-X1** | 2 | 2 | 0 | 0 | 4 |
| **NF-YC** | 2 | 1 | 1 | 0 | 4 |
| **CPP** | 2 | 1 | 0 | 0 | 3 |
| **RAV** | 0 | 1 | 1 | 1 | 3 |
| **BES1** | 0 | 0 | 2 | 0 | 2 |
| **HB-PHD** | 2 | 0 | 0 | 0 | 2 |
| **STAT** | 1 | 1 | 0 | 0 | 2 |
| **LFY** | 1 | 0 | 0 | 0 | 1 |
| **LSD** | 1 | 0 | 0 | 0 | 1 |
| **VOZ** | 1 | 0 | 0 | 0 | 1 |
