# Supplementary material for: De Novo Assembled Wheat Transcriptomes Delineate Differentially Expressed Host Genes in Response to Leaf Rust Infection
Source: PLoS One. 2016 Feb 3;11(2):e0148453. doi: 10.1371/journal.pone.0148453 (PMC4739524; doi:10.1371/journal.pone.0148453)
Supplement: S3 Table — (DOC) [file pone.0148453.s016.doc]

**S3 Table : List of the contigs having hits in the PRGdb database**

| **Query** | | **Number of hits** | **Lowest E-value** | | | | **Accession (E-value)** | | | | **Description** | | | |
| --- | --- | --- | --- | --- | --- | --- | --- | --- | --- | --- | --- | --- | --- | --- |
| SAGE1contig_1646 | | 100 | 0 | | | | PRGDB00050098 | | | | casein kinase | | | |
| SAGE1contig_4987 | | 100 | 5.27E-22 | | | | PRGDB00189558 | | | | EDR1,Ethylene-responsive protein kinase Le-CTR1 | | | |
| SAGE1contig_3898 | | 393 | 0 | | | | PRGDB00077471 | | | | L domain-like,LRR | | | |
| SAGE1contig_2309 | | 11 | 1.10E-43 | | | | PRGDB00192946 | | | | LRR | | | |
| SAGE1contig_6098 | | 12 | 1.97E-13 | | | | PRGDB00215267 | | | | LRR, Ser-Thr Kinase | | | |
| SAGE1contig_4677 | | 6 | 1.08E-16 | | | | PRGDB00077895 | | | | Mlo Mlo-related protein | | | |
| SAGE1contig_5184 | | 45 | 6.36E-67 | | | | PRGDB00189838 | | | | NB-ARC,NBS | | | |
| SAGE1contig_4309 | | 19 | 2.35E-07 | | | | PRGDB00050359 | | | | NB-ARC,NBS | | | |
| SAGE1contig_5459 | | 12 | 4.56E-11 | | | | PRGDB00198914 | | | | NBS, LRR | | | |
| SAGE1contig_1111 | | 10 | 1.48E-08 | | | | PRGDB00198784 | | | | NBS, LRR | | | |
| SAGE1contig_3430 | | 22 | 3.45E-12 | | | | PRGDB00205815 | | | | NBS, Ser-Thr Kinase | | | |
| SAGE1contig_233 | | 200 | 0 | | | | PRGDB00076605 | | | | Protein kinase-like | | | |
| SAGE1contig_6359 | | 112 | 0 | | | | PRGDB00193713 | | | | Protein kinase-like | | | |
| SAGE1contig_1867 | | 100 | 1.26E-50 | | | | PRGDB00197489 | | | | Protein kinase-like | | | |
| SAGE1contig_4740 | | 100 | 1.12E-23 | | | | PRGDB00194070 | | | | Protein kinase-like | | | |
| SAGE1contig_5991 | | 100 | 3.55E-170 | | | | PRGDB00077771 | | | | Protein kinase-like | | | |
| SAGE1contig_6668 | | 100 | 6.70E-68 | | | | PRGDB00070086 | | | | Protein kinase-like | | | |
| SAGE1contig_6144 | | 35 | 8.39E-56 | | | | PRGDB00204220 | | | | Protein kinase-like | | | |
| SAGE1contig_2599 | | 13 | 8.99E-38 | | | | PRGDB00192986 | | | | Protein kinase-like | | | |
| SAGE1contig_4405 | | 7 | 4.17E-105 | | | | PRGDB00215424 | | | | Protein kinase-like | | | |
| SAGE1contig_6530 | | 5 | 1.06E-24 | | | | PRGDB00206979 | | | | Protein kinase-like | | | |
| SAGE1contig_6647 | | 5 | 6.78E-136 | | | | PRGDB00199281 | | | | Protein kinase-like | | | |
| SAGE1contig_6719 | | 5 | 2.84E-16 | | | | PRGDB00216603 | | | | Protein kinase-like | | | |
| SAGE1contig_5635 | | 130 | 0 | | | | PRGDB00193011 | | | | Protein kinase-like | | | |
| SAGE1contig_2351 | | 100 | 1.30E-29 | | | | PRGDB00077899 | | | | Protein kinase-like | | | |
| SAGE1contig_2537 | | 100 | 1.19E-95 | | | | PRGDB00199870 | | | | Protein kinase-like | | | |
| SAGE1contig_3614 | | 100 | 0 | | | | PRGDB00192904 | | | | RAG1-ACTIVATING PROTEIN 1 | | | |
| SAGE1contig_7023 | | 12 | 2.46E-12 | | | | PRGDB00189464 | | | | Ser-Thr Kinase, LRR | | | |
| SAGE1contig_1700 | | 51 | 6.80E-60 | | | | PRGDB00213743 | | | | Toll/Interleukin receptor TIR domain | | | |
| SAGE1contig_3878 | | 13 | 1.28E-161 | | | | PRGDB00050102 | | | | transposon-like protein | | | |
| SAGE1contig_1143 | | 68 | 1.61E-37 | | | | PRGDB00050952 | | | | abscisic acid insensitive protein | | | |
| SAGE1contig_295 | | 67 | 2.27E-39 | | | | PRGDB00050952 | | | | abscisic acid insensitive protein | | | |
| SAGE1contig_1112 | | 46 | 3.09E-38 | | | | PRGDB00050952 | | | | abscisic acid insensitive protein | | | |
| SAGE1contig_860 | | 199 | 0 | | | | PRGDB00181834 | | | | ACT-like protein tyrosine kinase family protein | | | |
| SAGE1contig_6378 | | 140 | 2.23E-125 | | | | PRGDB00204312 | | | | Adenine nucleotide alpha hydrolases-like | | | |
| SAGE1contig_6196 | | 5 | 8.83E-23 | | | | PRGDB00177605 | | | | ARF GTPase-activating protein | | | |
| SAGE1contig_2775 | | 100 | 1.60E-159 | | | | PRGDB00189437 | | | | ATMRK_like,Ser-thr,PROTEIN KINASE ATMRK1 | | | |
| SAGE1contig_5628 | | 21 | 2.67E-14 | | | | PRGDB00069909 | | | | ATP-BINDING CASSETTE TRANSPORTER | | | |
| SAGE1contig_1005 | | 15 | 9.35E-10 | | | | PRGDB00077794 | | | | ATP-BINDING CASSETTE TRANSPORTER | | | |
| SAGE1contig_2639 | | 11 | 5.23E-21 | | | | PRGDB00061511 | | | | BrTNL10 disease resistance protein gene | | | |
| SAGE1contig_3965 | | 100 | 2.23E-55 | | | | PRGDB00050098 | | | | casein kinase | | | |
| SAGE1contig_5141 | | 10 | 8.60E-28 | | | | PRGDB00050098 | | | | casein kinase | | | |
| SAGE1contig_5132 | | 6 | 2.56E-16 | | | | PRGDB00050098 | | | | casein kinase | | | |
| SAGE1contig_5385 | | 100 | 0 | | | | PRGDB00077514 | | | | CINNAMOYL-COA REDUCTASE | | | |
| SAGE1contig_6976 | | 100 | 3.70E-171 | | | | PRGDB00193022 | | | | CINNAMOYL-COA REDUCTASE | | | |
| SAGE1contig_34 | | 84 | 6.63E-09 | | | | PRGDB00078801 | | | | CINNAMOYL-COA REDUCTASE | | | |
| SAGE1contig_2187 | | 13 | 5.51E-45 | | | | PRGDB00206825 | | | | Concanavalin A-like lectins/glucanases | | | |
| SAGE1contig_2609 | | 13 | 9.21E-42 | | | | PRGDB00206825 | | | | Concanavalin A-like lectins/glucanases | | | |
| SAGE1contig_5639 | | 5 | 4.14E-21 | | | | PRGDB00188382 | | | | cyclin-dependent kinase C-2-like | | | |
| SAGE1contig_5709 | | 5 | 4.91E-12 | | | | PRGDB00192427 | | | | Cysteine-rich receptor-like protein kinase | | | |
| SAGE1contig_4860 | | 105 | 4.18E-85 | | | | PRGDB00147227 | | | | cysteine-rich RLK (RECEPTOR-like protein kinase) | | | |
| SAGE1contig_2205 | | 50 | 1.49E-55 | | | | PRGDB00195789 | | | | DIMETHYLANILINE MONOOXYGENASE | | | |
| SAGE1contig_4452 | | 5 | 1.16E-07 | | | | PRGDB00213057 | | | | DISEASE RESISTANCE PROTEIN (TIR-NBS-LRR CLASS) | | | |
| SAGE1contig_2138 | | 16 | 2.83E-43 | | | | PRGDB00167870 | | | | Disease resistance protein (TIR-NBS-LRR class) family | | | |
| SAGE1contig_2261 | | 6 | 1.31E-18 | | | | PRGDB00062348 | | | | E3 ligase | | | |
| SAGE1contig_7018 | | 5 | 2.40E-10 | | | | PRGDB00177064 | | | | Flavin-binding monooxygenase family protein | | | |
| SAGE1contig_6078 | | 29 | 0 | | | | PRGDB00206824 | | | | GAG/POL/ENV POLYPROTEIN | | | |
| SAGE1contig_1511 | | 5 | 1.61E-93 | | | | PRGDB00171515 | | | | galacturonosyltransferase 1 | | | |
| SAGE1contig_5750 | | 101 | 1.01E-36 | | | | PRGDB00077578 | | | | GLYCOGEN SYNTHASE KINASE-3 ALPHA | | | |
| SAGE1contig_824 | | 100 | 0 | | | | PRGDB00208027 | | | | GLYCOGEN SYNTHASE KINASE-3 ALPHA | | | |
| SAGE1contig_2241 | | 9 | 6.06E-167 | | | | PRGDB00152926 | | | | importin alpha isoform 2 | | | |
| SAGE1contig_5868 | | 50 | 3.69E-09 | | | | PRGDB00166690 | | | | Integrin-linked protein kinase family | | | |
| SAGE1contig_5823 | | 13 | 1.31E-148 | | | | PRGDB00189575 | | | | IRE1P-RELATED | | | |
| SAGE1contig_4089 | | 972 | 1.82E-146 | | | | PRGDB00192883 | | | | L domain-like,LRR | | | |
| SAGE1contig_2717 | | 686 | 0 | | | | PRGDB00193062 | | | | L domain-like,LRR | | | |
| SAGE1contig_6529 | | 115 | 1.02E-52 | | | | PRGDB00189281 | | | | L domain-like,LRR | | | |
| SAGE1contig_4542 | | 100 | 2.87E-17 | | | | PRGDB00189540 | | | | L domain-like,LRR | | | |
| SAGE1contig_3436 | | 19 | 6.89E-12 | | | | PRGDB00077654 | | | | L domain-like,LRR | | | |
| SAGE1contig_5424 | | 100 | 1.76E-93 | | | | PRGDB00146654 | | | | Leucine-rich repeat transmembrane protein kinase protein | | | |
| SAGE1contig_4604 | | 212 | 5.90E-19 | | | | PRGDB00204027 | | | | LEUCINE-RICH REPEAT-CONTAINING PROTEIN | | | |
| SAGE1contig_3651 | | 206 | 1.52E-19 | | | | PRGDB00204028 | | | | LEUCINE-RICH REPEAT-CONTAINING PROTEIN | | | |
| SAGE1contig_6827 | | 151 | 1.41E-16 | | | | PRGDB00203969 | | | | LEUCINE-RICH REPEAT-CONTAINING PROTEIN | | | |
| SAGE1contig_3800 | | 105 | 0 | | | | PRGDB00078071 | | | | LEUCINE-RICH REPEAT-CONTAINING PROTEIN | | | |
| SAGE1contig_4026 | | 102 | 0 | | | | PRGDB00189767 | | | | LEUCINE-RICH REPEAT-CONTAINING PROTEIN | | | |
| SAGE1contig_300 | | 100 | 2.39E-64 | | | | PRGDB00192939 | | | | LEUCINE-RICH REPEAT-CONTAINING PROTEIN | | | |
| SAGE1contig_3985 | | 100 | 0 | | | | PRGDB00078071 | | | | LEUCINE-RICH REPEAT-CONTAINING PROTEIN | | | |
| SAGE1contig_1747 | | 60 | 1.86E-09 | | | | PRGDB00189633 | | | | LEUCINE-RICH REPEAT-CONTAINING PROTEIN | | | |
| SAGE1contig_5250 | | 33 | 5.77E-12 | | | | PRGDB00204203 | | | | LEUCINE-RICH REPEAT-CONTAINING PROTEIN | | | |
| SAGE1contig_4676 | | 29 | 1.26E-20 | | | | PRGDB00078033 | | | | LEUCINE-RICH REPEAT-CONTAINING PROTEIN | | | |
| SAGE1contig_5773 | | 23 | 9.60E-56 | | | | PRGDB00193055 | | | | LEUCINE-RICH REPEAT-CONTAINING PROTEIN | | | |
| SAGE1contig_25 | | 22 | 2.68E-14 | | | | PRGDB00077677 | | | | LEUCINE-RICH REPEAT-CONTAINING PROTEIN | | | |
| SAGE1contig_4520 | | 21 | 1.02E-13 | | | | PRGDB00077677 | | | | LEUCINE-RICH REPEAT-CONTAINING PROTEIN | | | |
| SAGE1contig_2405 | | 15 | 2.17E-12 | | | | PRGDB00199494 | | | | LEUCINE-RICH REPEAT-CONTAINING PROTEIN | | | |
| SAGE1contig_4612 | | 17 | 7.81E-16 | | | | PRGDB00208199 | | | | LEUCINE-RICH REPEAT-CONTAINING PROTEIN | | | |
| SAGE1contig_1276 | | 7 | 2.54E-10 | | | | PRGDB00077677 | | | | LEUCINE-RICH REPEAT-CONTAINING PROTEIN | | | |
| SAGE1contig_3239 | | 100 | 0 | | | | PRGDB00189306 | | | | LONGEVITY ASSURANCE FACTOR 1 (LAG1) | | | |
| SAGE1contig_581 | | 11 | 4.12E-29 | | | | PRGDB00206921 | | | | LRR | | | |
| SAGE1contig_4268 | | 8 | 7.10E-60 | | | | PRGDB00206897 | | | | LRR | | | |
| SAGE1contig_3506 | | 89 | 2.36E-17 | | | | PRGDB00171841 | | | | LRR and NB-ARC domains-containing disease resistance protein | | | |
| SAGE1contig_140 | | 16 | 1.07E-15 | | | | PRGDB00146686 | | | | LRR and NB-ARC domains-containing disease resistance protein | | | |
| SAGE1contig_3390 | | 9 | 1.69E-61 | | | | PRGDB00068820 | | | | LRR, NBS | | | |
| SAGE1contig_4928 | | 5 | 1.07E-111 | | | | PRGDB00183655 | | | | Major facilitator superfamily protein | | | |
| SAGE1contig_6542 | | 296 | 0 | | | | PRGDB00193806 | | | | Malectin_like,Carbohydrate-binding protein of the ER,Protein kinase-like | | | |
| SAGE1contig_1089 | | 77 | 1.79E-65 | | | | PRGDB00147385 | | | | mitogen-activated protein kinase kinase kinase 3 | | | |
| SAGE1contig_5516 | | 53 | 1.08E-41 | | | | PRGDB00145024 | | | | MSP domain containing protein | | | |
| SAGE1contig_4134 | | 102 | 3.44E-145 | | | | PRGDB00189629 | | | | NAD DEPENDENT EPIMERASE/DEHYDRATASE | | | |
| SAGE1contig_1154 | | 101 | 7.20E-142 | | | | PRGDB00193105 | | | | NAD DEPENDENT EPIMERASE/DEHYDRATASE | | | |
| SAGE1contig_5235 | | 101 | 0 | | | | PRGDB00193076 | | | | NAD DEPENDENT EPIMERASE/DEHYDRATASE | | | |
| SAGE1contig_4933 | | 120 | 0 | | | | PRGDB00180772 | | | | NB-ARC domain-containing disease resistance protein | | | |
| SAGE1contig_2364 | | 77 | 2.19E-10 | | | | PRGDB00180117 | | | | NB-ARC domain-containing disease resistance protein | | | |
| SAGE1contig_3196 | | 12 | 9.34E-07 | | | | PRGDB00180117 | | | | NB-ARC domain-containing disease resistance protein | | | |
| SAGE1contig_3007 | | 7 | 1.83E-16 | | | | PRGDB00165191 | | | | NB-ARC domain-containing disease resistance protein | | | |
| SAGE1contig_2314 | | 6 | 5.65E-15 | | | | PRGDB00183740 | | | | NB-ARC domain-containing disease resistance protein | | | |
| SAGE1contig_1266 | | 5 | 4.45E-06 | | | | PRGDB00146989 | | | | NB-ARC domain-containing disease resistance protein | | | |
| SAGE1contig_6923 | | 5 | 4.97E-07 | | | | PRGDB00146989 | | | | NB-ARC domain-containing disease resistance protein | | | |
| SAGE1contig_4526 | | 68 | 3.89E-43 | | | | PRGDB00150234 | | | | protein kinase family protein / protein phosphatase 2C ( PP2C) family protein | | | |
| SAGE1contig_556 | | 62 | 3.99E-36 | | | | PRGDB00142096 | | | | protein kinase family protein / protein phosphatase 2C ( PP2C) family protein | | | |
| SAGE1contig_3179 | | 218 | 5.27E-11 | | | | PRGDB00182659 | | | | Protein kinase superfamily protein | | | |
| SAGE1contig_1811 | | 81 | 1.33E-66 | | | | PRGDB00183826 | | | | Protein kinase superfamily protein | | | |
| SAGE1contig_6123 | | 200 | 2.64E-166 | | | | PRGDB00178056 | | | | Protein kinase superfamily protein with octicosapeptide/Phox/Bem1p domain | | | |
| SAGE1contig_1873 | | 213 | 3.20E-100 | | | | PRGDB00192858 | | | | Protein kinase-like | | | |
| SAGE1contig_1314 | | 209 | 0 | | | | PRGDB00193037 | | | | Protein kinase-like | | | |
| SAGE1contig_5837 | | 206 | 0 | | | | PRGDB00193245 | | | | Protein kinase-like | | | |
| SAGE1contig_3864 | | 201 | 0 | | | | PRGDB00077444 | | | | Protein kinase-like | | | |
| SAGE1contig_6344 | | 157 | 1.85E-21 | | | | PRGDB00197524 | | | | Protein kinase-like | | | |
| SAGE1contig_3886 | | 104 | 2.29E-131 | | | | PRGDB00187910 | | | | Protein kinase-like | | | |
| SAGE1contig_1948 | | 102 | 1.98E-28 | | | | PRGDB00189626 | | | | Protein kinase-like | | | |
| SAGE1contig_3047 | | 101 | 1.38E-128 | | | | PRGDB00076412 | | | | Protein kinase-like | | | |
| SAGE1contig_4864 | | 101 | 1.07E-114 | | | | PRGDB00192902 | | | | Protein kinase-like | | | |
| SAGE1contig_6000 | | 101 | 0 | | | | PRGDB00194358 | | | | Protein kinase-like | | | |
| SAGE1contig_2129 | | 100 | 6.35E-20 | | | | PRGDB00204420 | | | | Protein kinase-like | | | |
| SAGE1contig_2409 | | 100 | 1.33E-172 | | | | PRGDB00189521 | | | | Protein kinase-like | | | |
| SAGE1contig_3141 | | 100 | 2.42E-145 | | | | PRGDB00192970 | | | | Protein kinase-like | | | |
| SAGE1contig_3365 | | 100 | 2.96E-93 | | | | PRGDB00192844 | | | | Protein kinase-like | | | |
| SAGE1contig_6393 | | 100 | 8.27E-44 | | | | PRGDB00216033 | | | | Protein kinase-like | | | |
| SAGE1contig_6997 | | 100 | 1.26E-27 | | | | PRGDB00078018 | | | | Protein kinase-like | | | |
| SAGE1contig_4378 | | 90 | 6.22E-30 | | | | PRGDB00077875 | | | | Protein kinase-like | | | |
| SAGE1contig_2533 | | 77 | 1.19E-66 | | | | PRGDB00192899 | | | | Protein kinase-like | | | |
| SAGE1contig_4279 | | 72 | 1.70E-31 | | | | PRGDB00193352 | | | | Protein kinase-like | | | |
| SAGE1contig_2655 | | 51 | 7.26E-121 | | | | PRGDB00077754 | | | | Protein kinase-like | | | |
| SAGE1contig_2924 | | 33 | 5.85E-21 | | | | PRGDB00189694 | | | | Protein kinase-like | | | |
| SAGE1contig_1621 | | 32 | 1.41E-22 | | | | PRGDB00192888 | | | | Protein kinase-like | | | |
| SAGE1contig_3891 | | 5 | 2.79E-30 | | | | PRGDB00201647 | | | | Protein kinase-like | | | |
| SAGE1contig_5195 | | 5 | 2.04E-07 | | | | PRGDB00193263 | | | | Protein kinase-like | | | |
| SAGE1contig_2154 | | 13 | 6.95E-27 | | | | PRGDB00189485 | | | | Protein kinase-like domain | | | |
| SAGE1contig_7072 | | 6 | 4.65E-45 | | | | PRGDB00072236 | | | | Protein kinase-like domain | | | |
| SAGE1contig_2189 | | 5 | 2.03E-16 | | | | PRGDB00074158 | | | | Protein kinase-like domain | | | |
| SAGE1contig_4844 | | 100 | 0 | | | | PRGDB00069993 | | | | Protein kinase-like,L domain-like,LRR | | | |
| SAGE1contig_524 | | 229 | 4.25E-59 | | | | PRGDB00193155 | | | | RAG1-ACTIVATING PROTEIN 1 | | | |
| SAGE1contig_455 | | 100 | 0 | | | | PRGDB00193143 | | | | RAG1-ACTIVATING PROTEIN 1 | | | |
| SAGE1contig_5274 | | 100 | 0 | | | | PRGDB00193139 | | | | RAG1-ACTIVATING PROTEIN 1 | | | |
| SAGE1contig_6538 | | 100 | 0 | | | | PRGDB00192904 | | | | RAG1-ACTIVATING PROTEIN 1 | | | |
| SAGE1contig_1615 | | 41 | 2.21E-49 | | | | PRGDB00049679 | | | | receptor kinase-like protein, family member D, and retrofit (gag/pol) genes | | | |
| SAGE1contig_6829 | | 100 | 2.56E-19 | | | | PRGDB00146031 | | | | receptor lectin kinase | | | |
| SAGE1contig_1142 | | 100 | 6.49E-42 | | | | PRGDB00062448 | | | | receptor-like kinase gene | | | |
| SAGE1contig_6376 | | 39 | 3.26E-39 | | | | PRGDB00206880 | | | | RNI-like,LRR | | | |
| SAGE1contig_6949 | | 11 | 2.49E-14 | | | | PRGDB00209576 | | | | RNI-like,LRR | | | |
| SAGE1contig_2134 | | 48 | 4.32E-23 | | | | PRGDB00214649 | | | | Serine/threonine phosphatases | | | |
| SAGE1contig_2704 | | 26 | 9.41E-164 | | | | PRGDB00193815 | | | | Serine/Threonine protein kinases | | | |
| SAGE1contig_4734 | | 5 | 3.09E-11 | | | | PRGDB00213544 | | | | Ser-Thr Kinase, NBS | | | |
| SAGE1contig_5858 | | 100 | 0 | | | | PRGDB00073972 | | | | shaggy-related protein kinase 3 | | | |
| SAGE1contig_5675 | | 103 | 5.10E-20 | | | | PRGDB00147456 | | | | S-locus lectin protein kinase family protein | | | |
| SAGE1contig_147 | | 100 | 7.83E-47 | | | | PRGDB00069975 | | | | SNF1-related protein kinase | | | |
| SAGE1contig_6249 | | 107 | 0 | | | | PRGDB00063197 | | | | STEROIDOGENIC ACUTE REGULATORY PROTEIN | | | |
| SAGE1contig_2420 | | 100 | 9.39E-125 | | | | PRGDB00063199 | | | | STEROIDOGENIC ACUTE REGULATORY PROTEIN | | | |
| SAGE1contig_4873 | | 100 | 2.92E-59 | | | | PRGDB00063780 | | | | STEROIDOGENIC ACUTE REGULATORY PROTEIN | | | |
| SAGE1contig_817 | | 16 | 3.53E-21 | | | | PRGDB00163932 | | | | thioredoxin H-type 1 | | | |
| SAGE1contig_3953 | | 16 | 5.29E-17 | | | | PRGDB00163932 | | | | thioredoxin H-type 1 | | | |
| SAGE1contig_4912 | | 16 | 6.56E-19 | | | | PRGDB00163932 | | | | thioredoxin H-type 1 | | | |
| SAGE1contig_6177 | | 8 | 3.25E-94 | | | | PRGDB00186672 | | | | TIR-NBS-LRR type disease resistance protein | | | |
| SAGE1contig_4052 | | 7 | 4.78E-09 | | | | PRGDB00186556 | | | | TMV resistance protein N-like | | | |
| SAGE1contig_2780 | | 38 | 1.21E-26 | | | | PRGDB00217904 | | | | Toll/Interleukin receptor TIR domain | | | |
| SAGE1contig_6979 | | 104 | 4.63E-111 | | | | PRGDB00213743 | | | | Toll/interleukin-1 receptor homology (TIR) domain | | | |
| SAGE1contig_6531 | | 100 | 0 | | | | PRGDB00213743 | | | | Toll/interleukin-1 receptor homology (TIR) domain | | | |
| SAGE1contig_3101 | | 11 | 2.05E-19 | | | | PRGDB00181316 | | | | U-box domain-containing protein kinase family protein | | | |
| SAGE1contig_4535 | | 53 | 4.86E-50 | | | | PRGDB00061915 | | | | WRKY disease resistance protein mRNA | | | |
| SAGE2contig_4830 | | 44 | | 3.72E-16 | | | PRGDB00189505 | | | | | | | GNK2,Protein kinase-like |
| SAGE2contig_562 | | 559 | | 0 | | | PRGDB00193018 | | | | | | | L domain-like,LRR |
| SAGE2contig_798 | | 100 | | 1.72E-151 | | | PRGDB00193130 | | | | | | | L domain-like,LRR |
| SAGE2contig_1617 | | 27 | | 2.20E-19 | | | PRGDB00216326 | | | | | | | L domain-like,LRR |
| SAGE2contig_2620 | | 5 | | 6.09E-33 | | | PRGDB00078126 | | | | | | | L domain-like,LRR |
| SAGE2contig_625 | | 7 | | 3.35E-33 | | | PRGDB00177091 | | | | | | | NAC domain containing protein 17 |
| SAGE2contig_5439 | | 155 | | 4.75E-15 | | | PRGDB00203969 | | | | | | | P-loop containing nucleoside triphosphate hydrolases |
| SAGE2contig_109 | | 213 | | 2.88E-100 | | | PRGDB00192858 | | | | | | | Protein kinase-like |
| SAGE2contig_5735 | | 210 | | 1.98E-75 | | | PRGDB00189626 | | | | | | | Protein kinase-like |
| SAGE2contig_3988 | | 174 | | 1.02E-21 | | | PRGDB00192970 | | | | | | | Protein kinase-like |
| SAGE2contig_566 | | 104 | | 2.33E-18 | | | PRGDB00078018 | | | | | | | Protein kinase-like |
| SAGE2contig_2873 | | 101 | | 3.85E-27 | | | PRGDB00192871 | | | | | | | Protein kinase-like |
| SAGE2contig_661 | | 100 | | 2.21E-36 | | | PRGDB00192898 | | | | | | | Protein kinase-like |
| SAGE2contig_2456 | | 100 | | 5.35E-44 | | | PRGDB00069783 | | | | | | | Protein kinase-like |
| SAGE2contig_3973 | | 74 | | 4.80E-21 | | | PRGDB00192999 | | | | | | | Protein kinase-like |
| SAGE2contig_4037 | | 34 | | 4.60E-09 | | | PRGDB00192940 | | | | | | | Protein kinase-like |
| SAGE2contig_2600 | | 32 | | 5.58E-23 | | | PRGDB00192888 | | | | | | | Protein kinase-like |
| SAGE2contig_2583 | | 6 | | 2.43E-17 | | | PRGDB00072421 | | | | | | | Protein kinase-like |
| SAGE2contig_2628 | | 5 | | 3.33E-27 | | | PRGDB00195590 | | | | | | | Protein kinase-like |
| SAGE2contig_4076 | | 49 | | 2.15E-71 | | | PRGDB00146665 | | | | | | | 3'-phosphoinositide-dependent protein kinase 1 |
| SAGE2contig_1110 | | 84 | | 4.53E-24 | | | PRGDB00078803 | | | | | | | ABC transporter transmembrane region |
| SAGE2contig_5579 | | 24 | | 0 | | | PRGDB00206824 | | | | | | | Acid proteases |
| SAGE2contig_5614 | | 101 | | 4.28E-135 | | | PRGDB00050098 | | | | | | | casein kinase |
| SAGE2contig_4330 | | 100 | | 4.33E-55 | | | PRGDB00050098 | | | | | | | casein kinase |
| SAGE2contig_5539 | | 100 | | 1.10E-64 | | | PRGDB00180262 | | | | | | | CBL-interacting protein kinase 8 |
| SAGE2contig_4996 | | 100 | | 0 | | | PRGDB00077514 | | | | | | | CINNAMOYL-COA REDUCTASE |
| SAGE2contig_1514 | | 12 | | 1.77E-07 | | | PRGDB00195149 | | | | | | | CINNAMOYL-COA REDUCTASE |
| SAGE2contig_390 | | 9 | | 4.11E-14 | | | PRGDB00172718 | | | | | | | Concanavalin A-like lectin protein kinase family protein |
| SAGE2contig_3673 | | 5 | | 1.21E-32 | | | PRGDB00206979 | | | | | | | Cytochrome b5-like heme/steroid binding domain |
| SAGE2contig_4794 | | 5 | | 3.57E-39 | | | PRGDB00200909 | | | | | | | Cytochrome P450 |
| SAGE2contig_2937 | | 6 | | 1.71E-18 | | | PRGDB00062348 | | | | | | | E3 ligase |
| SAGE2contig_2146 | | 204 | | 0 | | | PRGDB00193460 | | | | | | | EGF/Laminin, Protein kinase |
| SAGE2contig_1743 | | 100 | | 2.30E-56 | | | PRGDB00193769 | | | | | | | EGF/Laminin, Protein kinase |
| SAGE2contig_4247 | | 100 | | 5.81E-111 | | | PRGDB00193180 | | | | | | | EGF/Laminin, Protein kinase |
| SAGE2contig_5625 | | 5 | | 2.89E-16 | | | PRGDB00216603 | | | | | | | FAR1 DNA binding domain,Protein kinase-like |
| SAGE2contig_3134 | | 12 | | 2.06E-76 | | | PRGDB00172582 | | | | | | | heat shock cognate protein 70-1 |
| SAGE2contig_1782 | | 6 | | 2.96E-47 | | | PRGDB00173148 | | | | | | | Heat shock protein 70 |
| SAGE2contig_4547 | | 10 | | 7.45E-137 | | | PRGDB00152926 | | | | | | | importin alpha isoform 2 |
| SAGE2contig_6037 | | 358 | | 0 | | | PRGDB00078053 | | | | | | | L domain-like,LRR |
| SAGE2contig_1898 | | 218 | | 1.81E-160 | | | PRGDB00193813 | | | | | | | L domain-like,LRR |
| SAGE2contig_4026 | | 206 | | 2.53E-92 | | | PRGDB00192922 | | | | | | | L domain-like,LRR |
| SAGE2contig_5389 | | 171 | | 1.62E-60 | | | PRGDB00193774 | | | | | | | L domain-like,LRR |
| SAGE2contig_989 | | 103 | | 6.85E-118 | | | PRGDB00192856 | | | | | | | L domain-like,LRR |
| SAGE2contig_997 | | 101 | | 2.07E-62 | | | PRGDB00193081 | | | | | | | L domain-like,LRR |
| SAGE2contig_2850 | | 100 | | 3.71E-52 | | | PRGDB00193277 | | | | | | | L domain-like,LRR |
| SAGE2contig_5968 | | 100 | | 2.38E-17 | | | PRGDB00189540 | | | | | | | L domain-like,LRR |
| SAGE2contig_2514 | | 38 | | 3.35E-25 | | | PRGDB00216326 | | | | | | | L domain-like,LRR |
| SAGE2contig_1530 | | 35 | | 9.10E-23 | | | PRGDB00189305 | | | | | | | L domain-like,LRR |
| SAGE2contig_2044 | | 19 | | 2.40E-07 | | | PRGDB00050359 | | | | | | | LEUCINE-RICH REPEAT-CONTAINING PROTEIN |
| SAGE2contig_3148 | | 22 | | 1.98E-14 | | | PRGDB00077677 | | | | | | | LRR,LEUCINE-RICH REPEAT-CONTAINING PROTEIN |
| SAGE2contig_3351 | | 22 | | 1.75E-14 | | | PRGDB00077677 | | | | | | | LRR,LEUCINE-RICH REPEAT-CONTAINING PROTEIN |
| SAGE2contig_3366 | | 22 | | 9.52E-15 | | | PRGDB00077677 | | | | | | | LRR,LEUCINE-RICH REPEAT-CONTAINING PROTEIN |
| SAGE2contig_5123 | | 22 | | 1.55E-14 | | | PRGDB00077677 | | | | | | | LRR,LEUCINE-RICH REPEAT-CONTAINING PROTEIN |
| SAGE2contig_674 | | 20 | | 4.37E-14 | | | PRGDB00077677 | | | | | | | LRR,LEUCINE-RICH REPEAT-CONTAINING PROTEIN |
| SAGE2contig_33 | | 16 | | 1.32E-10 | | | PRGDB00077677 | | | | | | | LRR,LEUCINE-RICH REPEAT-CONTAINING PROTEIN |
| SAGE2contig_3418 | | 7 | | 2.36E-32 | | | PRGDB00072425 | | | | | | | LRR,LEUCINE-RICH REPEAT-CONTAINING PROTEIN |
| SAGE2contig_2717 | | 6 | | 5.89E-99 | | | PRGDB00197494 | | | | | | | MFS general substrate transporter |
| SAGE2contig_2633 | | 100 | | 5.65E-33 | | | PRGDB00150892 | | | | | | | mitogen-activated protein kinase 18 |
| SAGE2contig_2115 | | 50 | | 5.67E-35 | | | PRGDB00179316 | | | | | | | mitogen-activated protein kinase kinase kinase 3 |
| SAGE2contig_6059 | | 37 | | 2.42E-44 | | | PRGDB00193227 | | | | | | | Mlo-related protein |
| SAGE2contig_2281 | | 31 | | 5.28E-11 | | | PRGDB00204203 | | | | | | | NB-ARC |
| SAGE2contig_2888 | | 30 | | 6.43E-20 | | | PRGDB00189685 | | | | | | | NB-ARC |
| SAGE2contig_2530 | | 96 | | 3.15E-22 | | | PRGDB00171666 | | | | | | | NB-ARC domain-containing disease resistance protein |
| SAGE2contig_5318 | | 51 | | 7.89E-10 | | | PRGDB00180117 | | | | | | | NB-ARC domain-containing disease resistance protein |
| SAGE2contig_1129 | | 47 | | 1.75E-89 | | | PRGDB00147348 | | | | | | | NB-ARC domain-containing disease resistance protein |
| SAGE2contig_2003 | | 12 | | 9.34E-07 | | | PRGDB00180117 | | | | | | | NB-ARC domain-containing disease resistance protein |
| SAGE2contig_5064 | | 11 | | 3.05E-07 | | | PRGDB00146989 | | | | | | | NB-ARC domain-containing disease resistance protein |
| SAGE2contig_3998 | | 24 | | 2.75E-56 | | | PRGDB00193055 | | | | | | | NB-ARC, |
| SAGE2contig_6068 | | 10 | | 3.23E-08 | | | PRGDB00198784 | | | | | | | NB-ARC,NBS |
| SAGE2contig_5120 | | 273 | | 4.18E-30 | | | PRGDB00181537 | | | | | | | NBS-LRR disease resistance protein |
| SAGE2contig_5926 | | 215 | | 5.80E-18 | | | PRGDB00189672 | | | | | | | P-loop containing nucleoside triphosphate hydrolases |
| SAGE2contig_2232 | | 206 | | 1.29E-19 | | | PRGDB00204028 | | | | | | | P-loop containing nucleoside triphosphate hydrolases |
| SAGE2contig_2013 | | 200 | | 2.58E-20 | | | PRGDB00204027 | | | | | | | P-loop containing nucleoside triphosphate hydrolases |
| SAGE2contig_5952 | | 105 | | 0 | | | PRGDB00192939 | | | | | | | P-loop containing nucleoside triphosphate hydrolases |
| SAGE2contig_5005 | | 102 | | 0 | | | PRGDB00189767 | | | | | | | P-loop containing nucleoside triphosphate hydrolases |
| SAGE2contig_185 | | 101 | | 2.45E-45 | | | PRGDB00193031 | | | | | | | P-loop containing nucleoside triphosphate hydrolases |
| SAGE2contig_3067 | | 100 | | 2.42E-82 | | | PRGDB00078071 | | | | | | | P-loop containing nucleoside triphosphate hydrolases |
| SAGE2contig_4130 | | 100 | | 1.67E-53 | | | PRGDB00193988 | | | | | | | P-loop containing nucleoside triphosphate hydrolases |
| SAGE2contig_5713 | | 100 | | 0 | | | PRGDB00193191 | | | | | | | P-loop containing nucleoside triphosphate hydrolases |
| SAGE2contig_5132 | | 100 | | 5.55E-19 | | | PRGDB00145952 | | | | | | | Protein kinase superfamily protein |
| SAGE2contig_6270 | | 22 | | 6.65E-58 | | | PRGDB00183826 | | | | | | | Protein kinase superfamily protein |
| SAGE2contig_4863 | | 6 | | 2.72E-26 | | | PRGDB00171644 | | | | | | | Protein kinase superfamily protein |
| SAGE2contig_3213 | | 24 | | 2.52E-21 | | | PRGDB00077658 | | | | | | | Protein kinase, catalytic domain |
| SAGE2contig_352 | | 218 | | 3.47E-11 | | | PRGDB00182659 | | | | | | | Protein kinase-like |
| SAGE2contig_2559 | | 181 | | 0 | | | PRGDB00193014 | | | | | | | Protein kinase-like |
| SAGE2contig_994 | | 155 | | 1.30E-111 | | | PRGDB00077932 | | | | | | | Protein kinase-like |
| SAGE2contig_1088 | | 141 | | 0 | | | PRGDB00193713 | | | | | | | Protein kinase-like |
| SAGE2contig_5150 | | 120 | | 0 | | | PRGDB00193229 | | | | | | | Protein kinase-like |
| SAGE2contig_1802 | | 114 | | 0 | | | PRGDB00193711 | | | | | | | Protein kinase-like |
| SAGE2contig_3589 | | 105 | | 8.45E-157 | | | PRGDB00193200 | | | | | | | Protein kinase-like |
| SAGE2contig_5308 | | 103 | | 2.54E-177 | | | PRGDB00146281 | | | | | | | Protein kinase-like |
| SAGE2contig_2048 | | 101 | | 0 | | | PRGDB00189577 | | | | | | | Protein kinase-like |
| SAGE2contig_5698 | | 101 | | 9.84E-97 | | | PRGDB00203942 | | | | | | | Protein kinase-like |
| SAGE2contig_5711 | | 101 | | 0 | | | PRGDB00192968 | | | | | | | Protein kinase-like |
| SAGE2contig_1143 | | 100 | | 4.70E-32 | | | PRGDB00077928 | | | | | | | Protein kinase-like |
| SAGE2contig_1360 | | 100 | | 6.51E-87 | | | PRGDB00077736 | | | | | | | Protein kinase-like |
| SAGE2contig_2161 | | 100 | | 2.65E-169 | | | PRGDB00189521 | | | | | | | Protein kinase-like |
| SAGE2contig_3051 | | 100 | | 5.46E-37 | | | PRGDB00077614 | | | | | | | Protein kinase-like |
| SAGE2contig_4222 | | 100 | | 4.99E-76 | | | PRGDB00070086 | | | | | | | Protein kinase-like |
| SAGE2contig_4519 | | 100 | | 5.66E-64 | | | PRGDB00077914 | | | | | | | Protein kinase-like |
| SAGE2contig_4737 | | 100 | | 4.75E-48 | | | PRGDB00195590 | | | | | | | Protein kinase-like |
| SAGE2contig_5562 | | 100 | | 5.68E-18 | | | PRGDB00204420 | | | | | | | Protein kinase-like |
| SAGE2contig_93 | | 71 | | 2.52E-47 | | | PRGDB00206954 | | | | | | | Protein kinase-like |
| SAGE2contig_5933 | | 6 | | 4.86E-109 | | | PRGDB00206966 | | | | | | | Protein kinase-like |
| SAGE2contig_879 | | 5 | | 1.78E-36 | | | PRGDB00201647 | | | | | | | Protein kinase-like |
| SAGE2contig_1664 | | 5 | | 2.04E-07 | | | PRGDB00193263 | | | | | | | Protein kinase-like |
| SAGE2contig_1770 | | 50 | | 5.05E-131 | | | PRGDB00193815 | | | | | | | Protein kinase-like WD40 repeat-like |
| SAGE2contig_475 | | 32 | | 2.08E-09 | | | PRGDB00213421 | | | | | | | Protein phosphatase 2C-like |
| SAGE2contig_4223 | | 101 | | 5.44E-62 | | | PRGDB00070000 | | | | | | | putative lectin receptor-type protein kinase |
| SAGE2contig_448 | | 100 | | 0 | | | PRGDB00192904 | | | | | | | RAG1-ACTIVATING PROTEIN 1 |
| SAGE2contig_3158 | | 69 | | 0 | | | PRGDB00049679 | | | | | | | receptor kinase-like protein |
| SAGE2contig_809 | | 52 | | 2.08E-115 | | | PRGDB00049679 | | | | | | | receptor kinase-like protein |
| SAGE2contig_5096 | | 18 | | 2.22E-15 | | | PRGDB00049679 | | | | | | | receptor kinase-like protein |
| SAGE2contig_3065 | | 101 | | 2.38E-32 | | | PRGDB00146031 | | | | | | | receptor lectin kinase |
| SAGE2contig_3732 | | 137 | | 2.12E-39 | | | PRGDB00147462 | | | | | | | receptor-like kinase |
| SAGE2contig_1005 | | 6 | | 2.69E-87 | | | PRGDB00206897 | | | | | | | Rhamnogalacturonate lyase,LRR,Zinc finger, CCHC-type |
| SAGE2contig_1543 | | 9 | | 2.77E-33 | | | PRGDB00206900 | | | | | | | Ribonuclease H-like |
| SAGE2contig_767 | | 125 | | 1.21E-133 | | | PRGDB00050492 | | | | | | | Ribonuclease H-like domain |
| SAGE2contig_4094 | | 7 | | 7.81E-10 | | | PRGDB00183040 | | | | | | | RING/U-box superfamily protein |
| SAGE2contig_6162 | | 104 | | 1.29E-156 | | | PRGDB00193181 | | | | | | | RNI-like,LRR |
| SAGE2contig_5187 | | 101 | | 4.00E-44 | | | PRGDB00150820 | | | | | | | serine/threonine protein kinase 2 |
| SAGE2contig_6012 | | 101 | | 1.21E-68 | | | PRGDB00160549 | | | | | | | serine/threonine protein kinase 2 |
| SAGE2contig_3931 | | 100 | | 0 | | | PRGDB00189258 | | | | | | | SSF52058,LRR |
| SAGE2contig_4010 | | 61 | | 1.16E-51 | | | PRGDB00076381 | | | | | | | Thaumatin, pathogenesis-related |
| SAGE2contig_3860 | | 11 | | 6.78E-78 | | | PRGDB00186672 | | | | | | | TIR-NBS-LRR type disease resistance protein |
| SAGE2contig_338 | | 37 | | 8.38E-101 | | | PRGDB00213743 | | | | | | | Toll/Interleukin receptor TIR domain |
| SAGE2contig_847 | | 5 | | 1.74E-12 | | | PRGDB00214760 | | | | | | | Toll/Interleukin receptor TIR domain |
| SAGE2contig_4329 | | 71 | | 5.51E-141 | | | PRGDB00077829 | | | | | | | TPR-like |
| SAGE2contig_3598 | | 9 | | 3.57E-166 | | | PRGDB00050102 | | | | | | | transposon-like protein |
| SAGE2contig_5402 | | 49 | | 4.00E-40 | | | PRGDB00061915 | | | | | | | WRKY disease resistance protein |
| SAGE3contig_2662 | | 59 | | 1.64E-82 | | | | | PRGDB00146571 | | | | protein kinase family protein / protein phosphatase 2C ( PP2C) family protein | |
| SAGE3contig_3326 | | 5 | | 1.19E-32 | | | | | PRGDB00206979 | | | | Cytochrome b5-like heme/steroid binding domain | |
| SAGE3contig_684 | | 5 | | 1.31E-40 | | | | | PRGDB00200909 | | | | Cytochrome P450 | |
| SAGE3contig_284 | | 40 | | 8.15E-23 | | | | | PRGDB00189305 | | | | L domain-like,LRR | |
| SAGE3contig_1661 | | 35 | | 1.69E-48 | | | | | PRGDB00077486 | | | | L domain-like,LRR | |
| SAGE3contig_144 | | 17 | | 7.90E-13 | | | | | PRGDB00077654 | | | | L domain-like,LRR | |
| SAGE3contig_1106 | | 6 | | 1.04E-09 | | | | | PRGDB00077654 | | | | L domain-like,LRR | |
| SAGE3contig_1201 | | 100 | | 2.35E-108 | | | | | PRGDB00069956 | | | | Protein kinase-like | |
| SAGE3contig_3331 | | 100 | | 2.24E-172 | | | | | PRGDB00189521 | | | | Protein kinase-like | |
| SAGE3contig_3832 | | 39 | | 2.60E-39 | | | | | PRGDB00206880 | | | | RNI-like,LRR | |
| SAGE3contig_3188 | | 10 | | 1.42E-31 | | | | | PRGDB00078071 | | | | ,LEUCINE-RICH REPEAT-CONTAINING PROTEIN | |
| SAGE3contig_2371 | | 101 | | 2.17E-44 | | | | | PRGDB00078803 | | | | ABC transporter transmembrane region | |
| SAGE3contig_1454 | | 87 | | 1.53E-31 | | | | | PRGDB00078803 | | | | ABC transporter transmembrane region | |
| SAGE3contig_3473 | | 28 | | 8.74E-13 | | | | | PRGDB00079245 | | | | ABC-2 type transporter | |
| SAGE3contig_863 | | 199 | | 0 | | | | | PRGDB00147269 | | | | ACT-like protein tyrosine kinase family protein | |
| SAGE3contig_2978 | | 100 | | 6.38E-153 | | | | | PRGDB00193022 | | | | CINNAMOYL-COA REDUCTASE | |
| SAGE3contig_3983 | | 100 | | 0 | | | | | PRGDB00077514 | | | | CINNAMOYL-COA REDUCTASE | |
| SAGE3contig_1793 | | 73 | | 2.67E-09 | | | | | PRGDB00078801 | | | | CINNAMOYL-COA REDUCTASE | |
| SAGE3contig_471 | | 6 | | 1.05E-38 | | | | | PRGDB00216561 | | | | Cysteine proteinases | |
| SAGE3contig_1883 | | 5 | | 2.72E-25 | | | | | PRGDB00206979 | | | | Cytochrome b5-like heme/steroid binding domain | |
| SAGE3contig_1069 | | 16 | | 2.63E-43 | | | | | PRGDB00167870 | | | | Disease resistance protein (TIR-NBS-LRR class) family | |
| SAGE3contig_1052 | | 17 | | 1.18E-38 | | | | | PRGDB00192986 | | | | EGF/Laminin | |
| SAGE3contig_22 | | 22 | | 1.98E-14 | | | | | PRGDB00077677 | | | | FAMILY NOT NAMED,LRR,LEUCINE-RICH REPEAT-CONTAINING PROTEIN | |
| SAGE3contig_2673 | | 22 | | 1.20E-14 | | | | | PRGDB00077677 | | | | FAMILY NOT NAMED,LRR,LEUCINE-RICH REPEAT-CONTAINING PROTEIN | |
| SAGE3contig_4400 | | 22 | | 1.54E-14 | | | | | PRGDB00077677 | | | | FAMILY NOT NAMED,LRR,LEUCINE-RICH REPEAT-CONTAINING PROTEIN | |
| SAGE3contig_3419 | | 106 | | 4.63E-114 | | | | | PRGDB00188382 | | | | Glycine max cyclin-dependent kinase C-2-like | |
| SAGE3contig_1325 | | 12 | | 6.60E-32 | | | | | PRGDB00194821 | | | | Glycoside hydrolase, Protein kinase-like | |
| SAGE3contig_714 | | 104 | | 4.04E-23 | | | | | PRGDB00198969 | | | | Gnk2-homologous domain | |
| SAGE3contig_4189 | | 8 | | 0 | | | | | PRGDB00167710 | | | | heat shock cognate protein 70-1 | |
| SAGE3contig_3057 | | 7 | | 0 | | | | | PRGDB00167710 | | | | heat shock cognate protein 70-1 | |
| SAGE3contig_4461 | | 157 | | 7.13E-92 | | | | | PRGDB00193813 | | | | L domain-like,LRR | |
| SAGE3contig_4215 | | 102 | | 3.18E-79 | | | | | PRGDB00193132 | | | | L domain-like,LRR | |
| SAGE3contig_3774 | | 101 | | 0 | | | | | PRGDB00193130 | | | | L domain-like,LRR | |
| SAGE3contig_4330 | | 16 | | 7.28E-07 | | | | | PRGDB00077690 | | | | L domain-like,LRR | |
| SAGE3contig_926 | | 89 | | 2.28E-17 | | | | | PRGDB00171841 | | | | LRR and NB-ARC domains-containing disease resistance protein | |
| SAGE3contig_986 | | 50 | | 8.70E-34 | | | | | PRGDB00179316 | | | | mitogen-activated protein kinase kinase kinase 3 | |
| SAGE3contig_1399 | | 165 | | 3.59E-108 | | | | | PRGDB00193076 | | | | NAD DEPENDENT EPIMERASE/DEHYDRATASE | |
| SAGE3contig_3847 | | 102 | | 4.35E-99 | | | | | PRGDB00204013 | | | | NAD DEPENDENT EPIMERASE/DEHYDRATASE | |
| SAGE3contig_4406 | | 265 | | 0 | | | | | PRGDB00192843 | | | | NB-ARC | |
| SAGE3contig_2705 | | 151 | | 0 | | | | | PRGDB00180772 | | | | NB-ARC domain-containing disease resistance protein | |
| SAGE3contig_788 | | 6 | | 5.16E-15 | | | | | PRGDB00183740 | | | | NB-ARC domain-containing disease resistance protein | |
| SAGE3contig_1012 | | 5 | | 4.88E-06 | | | | | PRGDB00146989 | | | | NB-ARC domain-containing disease resistance protein | |
| SAGE3contig_1554 | | 100 | | 2.45E-121 | | | | | PRGDB00146670 | | | | Nodulin MtN3 family protein | |
| SAGE3contig_2419 | | 153 | | 1.96E-44 | | | | | PRGDB00063643 | | | | Pentatricopeptide repeat | |
| SAGE3contig_3239 | | 146 | | 2.45E-75 | | | | | PRGDB00070037 | | | | P-loop containing nucleoside triphosphate hydrolases | |
| SAGE3contig_2559 | | 56 | | 4.37E-51 | | | | | PRGDB00192982 | | | | P-loop containing nucleoside triphosphate hydrolases | |
| SAGE3contig_2776 | | 28 | | 3.42E-19 | | | | | PRGDB00189685 | | | | P-loop containing nucleoside triphosphate hydrolases | |
| SAGE3contig_3550 | | 15 | | 1.67E-84 | | | | | PRGDB00192920 | | | | P-loop containing nucleoside triphosphate hydrolases | |
| SAGE3contig_427 | | 100 | | 1.17E-142 | | | | | PRGDB00077446 | | | | PLP-dependent transferases | |
| SAGE3contig_3989 | | 59 | | 3.26E-113 | | | | | PRGDB00193815 | | | | Protein kinase | |
| SAGE3contig_1700 | | 67 | | 1.18E-40 | | | | | PRGDB00150234 | | | | protein kinase family protein / protein phosphatase 2C ( PP2C) family protein | |
| SAGE3contig_2229 | | 55 | | 4.42E-15 | | | | | PRGDB00155270 | | | | protein kinase family protein / protein phosphatase 2C ( PP2C) family protein | |
| SAGE3contig_4329 | | 218 | | 2.96E-11 | | | | | PRGDB00182659 | | | | Protein kinase superfamily protein | |
| SAGE3contig_421 | | 100 | | 5.24E-16 | | | | | PRGDB00184802 | | | | Protein kinase superfamily protein | |
| SAGE3contig_2389 | | 100 | | 5.11E-20 | | | | | PRGDB00145952 | | | | Protein kinase superfamily protein | |
| SAGE3contig_2136 | | 273 | | 4.34E-95 | | | | | PRGDB00217150 | | | | Protein kinase-like | |
| SAGE3contig_4563 | | 212 | | 3.05E-61 | | | | | PRGDB00189626 | | | | Protein kinase-like | |
| SAGE3contig_3803 | | 208 | | 0 | | | | | PRGDB00193245 | | | | Protein kinase-like | |
| SAGE3contig_2313 | | 188 | | 6.97E-17 | | | | | PRGDB00069740 | | | | Protein kinase-like | |
| SAGE3contig_2941 | | 107 | | 2.67E-49 | | | | | PRGDB00189626 | | | | Protein kinase-like | |
| SAGE3contig_4102 | | 102 | | 1.82E-72 | | | | | PRGDB00193995 | | | | Protein kinase-like | |
| SAGE3contig_322 | | 101 | | 3.35E-109 | | | | | PRGDB00077596 | | | | Protein kinase-like | |
| SAGE3contig_519 | | 100 | | 3.80E-44 | | | | | PRGDB00070086 | | | | Protein kinase-like | |
| SAGE3contig_565 | | 100 | | 3.96E-36 | | | | | PRGDB00077614 | | | | Protein kinase-like | |
| SAGE3contig_1648 | | 100 | | 0 | | | | | PRGDB00189513 | | | | Protein kinase-like | |
| SAGE3contig_2625 | | 100 | | 1.42E-27 | | | | | PRGDB00195697 | | | | Protein kinase-like | |
| SAGE3contig_2977 | | 100 | | 1.12E-89 | | | | | PRGDB00077644 | | | | Protein kinase-like | |
| SAGE3contig_3412 | | 100 | | 4.05E-56 | | | | | PRGDB00215446 | | | | Protein kinase-like | |
| SAGE3contig_3480 | | 100 | | 2.99E-64 | | | | | PRGDB00077536 | | | | Protein kinase-like | |
| SAGE3contig_4556 | | 100 | | 2.44E-70 | | | | | PRGDB00069817 | | | | Protein kinase-like | |
| SAGE3contig_4585 | | 21 | | 1.20E-12 | | | | | PRGDB00077683 | | | | Protein kinase-like | |
| SAGE3contig_3796 | | 14 | | 3.77E-09 | | | | | PRGDB00203993 | | | | Protein kinase-like | |
| SAGE3contig_2581 | | 5 | | 3.78E-38 | | | | | PRGDB00201647 | | | | Protein kinase-like | |
| SAGE3contig_2953 | | 100 | | 6.38E-18 | | | | | PRGDB00069740 | | | | Protein kinase-like domain | |
| SAGE3contig_333 | | 10 | | 3.37E-18 | | | | | PRGDB00214649 | | | | Protein phosphatase 2C-like | |
| SAGE3contig_922 | | 101 | | 0 | | | | | PRGDB00192904 | | | | RAG1-ACTIVATING PROTEIN 1 | |
| SAGE3contig_1480 | | 101 | | 6.81E-148 | | | | | PRGDB00192904 | | | | RAG1-ACTIVATING PROTEIN 1 | |
| SAGE3contig_252 | | 100 | | 0 | | | | | PRGDB00193143 | | | | RAG1-ACTIVATING PROTEIN 1 | |
| SAGE3contig_4288 | | 100 | | 0 | | | | | PRGDB00192904 | | | | RAG1-ACTIVATING PROTEIN 1 | |
| SAGE3contig_4568 | | 100 | | 0 | | | | | PRGDB00193129 | | | | RAG1-ACTIVATING PROTEIN 1 | |
| SAGE3contig_47 | | 102 | | 2.87E-119 | | | | | PRGDB00077538 | | | | RAG1-activating protein-1-related | |
| SAGE3contig_75 | | 6 | | 7.86E-46 | | | | | PRGDB00072236 | | | | SCP-like_extracellular CAP domain, | |
| SAGE3contig_2066 | | 23 | | 4.75E-11 | | | | | PRGDB00217738 | | | | Toll/Interleukin receptor TIR domain | |
| SAGE3contig_318 | | 5 | | 1.95E-12 | | | | | PRGDB00214760 | | | | Toll/Interleukin receptor TIR domain | |
| SAGE3contig_3998 | | 13 | | 6.52E-50 | | | | | PRGDB00170617 | | | | U-box domain-containing protein kinase family protein | |
| SAGE4contig_2781 | | 5 | | | | 1.33E-29 | | | PRGDB00062348 | | | E3 ligase | | |
| SAGE4contig_1736 | | 36 | | | | 2.30E-31 | | | PRGDB00187933 | | | Protein kinase-like | | |
| SAGE4contig_560 | | 87 | | | | 1.55E-31 | | | PRGDB00078803 | | | ABC transporter transmembrane region | | |
| SAGE4contig_2662 | | 31 | | | | 0 | | | PRGDB00206824 | | | Acid proteases | | |
| SAGE4contig_2943 | | 53 | | | | 2.07E-31 | | | PRGDB00077513 | | | ATP-BINDING CASSETTE TRANSPORTER PLANT | | |
| SAGE4contig_1486 | | 202 | | | | 7.60E-140 | | | PRGDB00050098 | | | casein kinase | | |
| SAGE4contig_2810 | | 100 | | | | 0 | | | PRGDB00077514 | | | CINNAMOYL-COA REDUCTASE | | |
| SAGE4contig_80 | | 6 | | | | 6.31E-37 | | | PRGDB00206829 | | | Concanavalin A-like lectins/glucanases | | |
| SAGE4contig_2255 | | 5 | | | | 1.70E-25 | | | PRGWK00001242 | | | Contributed R-Genes | | |
| SAGE4contig_1442 | | 105 | | | | 2.45E-82 | | | PRGDB00147227 | | | cysteine-rich RLK (RECEPTOR-like protein kinase) | | |
| SAGE4contig_1376 | | 143 | | | | 1.24E-08 | | | PRGDB00204000 | | | FAMILY NOT NAMED,LRR,LEUCINE-RICH REPEAT-  CONTAINING PROTEIN | | |
| SAGE4contig_3286 | | 68 | | | | 9.54E-20 | | | PRGDB00189505 | | | GNK2,Gnk2-homologous domain profile,  Protein kinase-like | | |
| SAGE4contig_1593 | | 1042 | | | | 0 | | | PRGDB00192952 | | | L domain-like,LRR | | |
| SAGE4contig_2635 | | 101 | | | | 0 | | | PRGDB00077983 | | | L domain-like,LRR | | |
| SAGE4contig_3248 | | 101 | | | | 0 | | | PRGDB00193130 | | | L domain-like,LRR | | |
| SAGE4contig_373 | | 11 | | | | 1.76E-43 | | | PRGDB00192946 | | | L domain-like,LRR | | |
| SAGE4contig_3079 | | 153 | | | | 2.17E-110 | | | PRGDB00061469 | | | Lr1 disease resistance protein | | |
| SAGE4contig_2055 | | 26 | | | | 1.41E-07 | | | PRGDB00193295 | | | NAD-dependent epimerase/dehydratase | | |
| SAGE4contig_2832 | | 151 | | | | 0 | | | PRGDB00180772 | | | NB-ARC domain-containing disease resistance  protein | | |
| SAGE4contig_918 | | 8 | | | | 6.16E-44 | | | PRGDB00078071 | | | NB-ARC,NBS | | |
| SAGE4contig_1232 | | 29 | | | | 1.23E-133 | | | PRGDB00193815 | | | Protein kinase | | |
| SAGE4contig_2285 | | 218 | | | | 5.27E-11 | | | PRGDB00182659 | | | Protein kinase superfamily protein | | |
| SAGE4contig_60 | | 361 | | | | 9.16E-94 | | | PRGDB00217150 | | | Protein kinase-like | | |
| SAGE4contig_540 | | 174 | | | | 9.15E-22 | | | PRGDB00192970 | | | Protein kinase-like | | |
| SAGE4contig_1997 | | 104 | | | | 2.48E-84 | | | PRGDB00193441 | | | Protein kinase-like | | |
| SAGE4contig_452 | | 100 | | | | 9.70E-51 | | | PRGDB00197489 | | | Protein kinase-like | | |
| SAGE4contig_2238 | | 100 | | | | 4.28E-40 | | | PRGDB00069783 | | | Protein kinase-like | | |
| SAGE4contig_3388 | | 100 | | | | 7.99E-173 | | | PRGDB00189521 | | | Protein kinase-like | | |
| SAGE4contig_2466 | | 32 | | | | 3.28E-22 | | | PRGDB00192888 | | | Protein kinase-like | | |
| SAGE4contig_2239 | | 6 | | | | 3.10E-10 | | | PRGDB00216603 | | | Protein kinase-like,FAR1 DNA binding domain | | |
| SAGE4contig_1086 | | 16 | | | | 2.07E-14 | | | PRGDB00049679 | | | receptor kinase-like protein | | |
| SAGE4contig_2319 | | 14 | | | | 0 | | | PRGDB00186672 | | | TIR-NBS-LRR type disease resistance protein | | |
| SAGE4contig_3325 | 17 | | | | 6.17E-17 | | | PRGDB00181316 | | U-box domain-containing protein kinase  family protein TPR-like | | | | |
|  | | | | | | |  | | | | | | | |
