# Supplementary material for: De Novo Assembled Wheat Transcriptomes Delineate Differentially Expressed Host Genes in Response to Leaf Rust Infection
Source: PLoS One. 2016 Feb 3;11(2):e0148453. doi: 10.1371/journal.pone.0148453 (PMC4739524; doi:10.1371/journal.pone.0148453)
Supplement: S4 Table — (DOC) [file pone.0148453.s017.doc]

**S4 Table**. List of the enriched GO terms showing the number of differentially upregulated contigs of S-M and S-PI

| **GO-Term** | **Description** | **Upregulated in S-M** | **Upregulated in S-PI** | **Enrichment P-value** |
| --- | --- | --- | --- | --- |
| **Cellular Component** |  |  |  |  |
| **GO:0005576** | extracellular region | 23 | 36 | 0.019 |
| **GO:0005623** | cell | 274 | 275 | 0.033 |
| **GO:0005622** | intracellular | 265 | 264 | 0.046 |
| **GO:0016020** | membrane | 47 | 52 | 0.179 |
| **GO:0030529** | ribonucleoprotein complex | 21 | 29 | 0.083 |
| **GO:0043229** | intracellular organelle | 233 | 249 | 0.007 |
| **GO:0032991** | macromolecular complex | 48 | 83 | 0.000 |
| **GO:0043234** | protein complex | 29 | 54 | 0.000 |
| **GO:0031982** | vesicle | 36 | 45 | 0.077 |
| **GO:0043227** | membrane-bounded organelle | 227 | 247 | 0.004 |
| **GO:0043228** | non-membrane-bounded organelle | 39 | 41 | 0.329 |
| **Biological Process** |  |  |  |  |
| **GO:0000003** | reproduction | 23 | 13 | 0.238 |
| **GO:0002376** | immune system process | 8 | 2 |  |
| **GO:0008152** | metabolic process | 192 | 212 | 0.005 |
| **GO:0006807** | nitrogen compound metabolic process | 76 | 73 | 0.436 |
| **GO:0006139** | nucleobase, nucleoside, nucleotide and nucleic acid metabolic process | 34 | 28 | 0.910 |
| **GO:0006259** | DNA metabolic process | 14 | 12 | 0.977 |
| **GO:0016070** | RNA metabolic process | 7 | 2 |  |
| **GO:0009308** | amine metabolic process | 15 | 20 | 0.179 |
| **GO:0009056** | catabolic process | 57 | 36 | 0.161 |
| **GO:0009058** | biosynthetic process | 62 | 91 | 0.001 |
| **GO:0009059** | macromolecule biosynthetic process | 15 | 26 | 0.023 |
| **GO:0019748** | secondary metabolic process | 2 | 4 |  |
| **GO:0043170** | macromolecule metabolic process | 76 | 85 | 0.072 |
| **GO:0010467** | gene expression | 33 | 26 | 0.780 |
| **GO:0006396** | RNA processing | 6 | 0 |  |
| **GO:0006412** | translation | 15 | 26 | 0.023 |
| **GO:0051604** | protein maturation | 12 | 0 | 0.001 |
| **GO:0019538** | protein metabolic process | 58 | 74 | 0.017 |
| **GO:0034960** | cellular biopolymer metabolic process | 64 | 85 | 0.005 |
| **GO:0043412** | biopolymer modification | 30 | 43 | 0.024 |
| **GO:0006082** | organic acid metabolic process | 15 | 20 | 0.179 |
| **GO:0006091** | generation of precursor metabolites and energy | 7 | 46 | 0.000 |
| **GO:0006790** | sulfur metabolic process | 2 | 11 | 0.005 |
| **GO:0015979** | photosynthesis | 9 | 61 | 0.000 |
| **GO:0051186** | cofactor metabolic process | 9 | 16 | 0.068 |
| **GO:0005975** | carbohydrate metabolic process | 45 | 56 | 0.051 |
| **GO:0006629** | lipid metabolic process | 27 | 18 | 0.427 |
| **GO:0019538** | protein metabolic process | 58 | 74 | 0.017 |
| **GO:0051604** | protein maturation | 12 | 0 | 0.001 |
| **GO:0009987** | cellular process | 159 | 211 | 0.000 |
| **GO:0006996** | organelle organization | 13 | 4 | 0.064 |
| **GO:0007010** | cytoskeleton organization | 6 | 2 |  |
| **GO:0051276** | chromosome organization | 7 | 2 |  |
| **GO:0007165** | signal transduction | 22 | 8 | 0.034 |
| **GO:0042254** | ribosome biogenesis | 0 | 6 |  |
| **GO:0051641** | cellular localization | 2 | 4 |  |
| **GO:0000902** | cell morphogenesis | 6 | 2 |  |
| **GO:0055085** | transmembrane transport | 17 | 33 | 0.004 |
| **GO:0010926** | anatomical structure formation | 15 | 13 | 0.952 |
| **GO:0016043** | cellular component organization | 23 | 14 | 0.325 |
| **GO:0051276** | chromosome organization | 7 | 2 |  |
| **GO:0032502** | developmental process | 44 | 37 | 0.972 |
| **GO:0007568** | aging | 5 | 8 | 0.256 |
| **GO:0021700** | developmental maturation | 3 | 5 |  |
| **GO:0043473** | pigmentation | 22 | 8 | 0.034 |
| **GO:0044085** | cellular component biogenesis | 13 | 17 | 0.234 |
| **GO:0006950** | response to stress | 84 | 90 | 0.113 |
| **GO:0051179** | localization | 52 | 55 | 0.245 |
| **GO:0006810** | transport | 52 | 55 | 0.245 |
| **GO:0015031** | protein transport | 2 | 4 |  |
| **GO:0007034** | vacuolar transport | 0 | 2 |  |
| **GO:0044403** | symbiosis, encompassing mutualism through parasitism | 1 | 4 |  |
| **GO:0065007** | biological regulation | 34 | 23 | 0.400 |
| **GO:0065008** | regulation of biological quality | 14 | 17 | 0.314 |
| **Molecular Function** |  |  |  |  |
| **GO:0003824** | catalytic activity | 191 | 180 | 0.284 |
| **GO:0005198** | structural molecule activity | 11 | 22 | 0.016 |
| **GO:0005215** | transporter activity | 21 | 33 | 0.024 |
| **GO:0005488** | binding | 171 | 167 | 0.173 |
| **GO:0030234** | enzyme regulator activity | 15 | 4 | 0.030 |
| **GO:0045182** | translation regulator activity | 5 | 7 | 0.386 |
| **GO:0060089** | molecular transducer activity | 1 | 3 |  |
