# Supplementary material for: De Novo Assembled Wheat Transcriptomes Delineate Differentially Expressed Host Genes in Response to Leaf Rust Infection
Source: PLoS One. 2016 Feb 3;11(2):e0148453. doi: 10.1371/journal.pone.0148453 (PMC4739524; doi:10.1371/journal.pone.0148453)
Supplement: S5 Table — (DOC) [file pone.0148453.s018.doc]

**S5 Table**. List of the enriched GO terms showing the number of differentially upregulated contigs of R-M and R-PI

| **GO-Term** | **Description** | **Upregulated in R-M** | **Upregulated in R-PI** | **Enrichment P-value** |
| --- | --- | --- | --- | --- |
| **Cellular Component** |  |  |  |  |
| **GO:0005576** | extracellular region | 31 | 8 | 0.037 |
| **GO:0005623** | cell | 275 | 65 | 0.000 |
| **GO:0005622** | intracellular | 268 | 58 | 0.000 |
| **GO:0016020** | membrane | 60 | 13 | 0.001 |
| **GO:0030312** | external encapsulating structure | 24 | 7 | 0.105 |
| **GO:0030529** | ribonucleoprotein complex | 19 | 5 | 0.109 |
| **GO:0044446** | intracellular organelle part | 24 | 3 | 0.006 |
| **GO:0043229** | intracellular organelle | 241 | 49 | 0.000 |
| **GO:0031974** | membrane-enclosed lumen | 21 | 2 | 0.005 |
| **GO:0032991** | macromolecular complex | 60 | 17 | 0.008 |
| **GO:0030529** | ribonucleoprotein complex | 19 | 5 | 0.109 |
| **GO:0043234** | protein complex | 42 | 12 | 0.028 |
| **GO:0043226** | organelle | 243 | 49 | 0.000 |
| **GO:0031982** | vesicle | 41 | 9 | 0.006 |
| **GO:0043227** | membrane-bounded organelle | 235 | 49 | 0.000 |
| **GO:0043228** | non-membrane-bounded organelle | 41 | 6 | 0.001 |
| **GO:0030312** | external encapsulating structure | 24 | 7 | 0.105 |
| **Biological Process** |  |  |  |  |
| **GO:0000003** | reproduction | 17 | 2 | 0.018 |
| **GO:0002376** | immune system process | 5 | 0 |  |
| **GO:0008152** | metabolic process | 187 | 39 | 0.000 |
| **GO:0006807** | nitrogen compound metabolic process | 79 | 17 | 0.000 |
| **GO:0006139** | nucleobase, nucleoside, nucleotide and nucleic acid metabolic process | 23 | 6 | 0.075 |
| **GO:0006259** | DNA metabolic process | 10 | 2 |  |
| **GO:0016070** | RNA metabolic process | 1 | 2 |  |
| **GO:0009308** | amine metabolic process | 15 | 6 | 0.448 |
| **GO:0034641** | cellular nitrogen compound metabolic process | 67 | 14 | 0.000 |
| **GO:0009056** | catabolic process | 46 | 7 | 0.000 |
| **GO:0009058** | biosynthetic process | 82 | 18 | 0.000 |
| **GO:0009059** | macromolecule biosynthetic process | 28 | 6 | 0.021 |
| **GO:0019748** | secondary metabolic process | 4 | 0 |  |
| **GO:0043170** | macromolecule metabolic process | 81 | 12 | 0.000 |
| **GO:0010467** | gene expression | 34 | 6 | 0.004 |
| **GO:0006412** | translation | 28 | 6 | 0.021 |
| **GO:0051604** | protein maturation | 6 | 0 |  |
| **GO:0043283** | biopolymer metabolic process | 81 | 12 | 0.000 |
| **GO:0019538** | protein metabolic process | 72 | 11 | 0.000 |
| **GO:0006082** | organic acid metabolic process | 15 | 6 | 0.448 |
| **GO:0006091** | generation of precursor metabolites and energy | 24 | 7 | 0.105 |
| **GO:0006790** | sulfur metabolic process | 6 | 1 |  |
| **GO:0015979** | photosynthesis | 16 | 11 | 0.645 |
| **GO:0051186** | cofactor metabolic process | 11 | 3 | 0.240 |
| **GO:0044238** | primary metabolic process | 141 | 27 | 0.000 |
| **GO:0005975** | carbohydrate metabolic process | 47 | 13 | 0.016 |
| **GO:0006629** | lipid metabolic process | 16 | 8 | 0.746 |
| **GO:0019538** | protein metabolic process | 72 | 11 | 0.000 |
| **GO:0009987** | cellular process | 190 | 46 | 0.000 |
| **GO:0006996** | organelle organization | 15 | 2 | 0.034 |
| **GO:0007010** | cytoskeleton organization | 5 | 2 |  |
| **GO:0007049** | cell cycle | 4 | 0 |  |
| **GO:0051276** | chromosome organization | 9 | 0 |  |
| **GO:0007165** | signal transduction | 13 | 5 | 0.440 |
| **GO:0016070** | RNA metabolic process | 1 | 2 |  |
| **GO:0055085** | transmembrane transport | 21 | 13 | 0.832 |
| **GO:0010926** | anatomical structure formation | 16 | 3 | 0.060 |
| **GO:0016043** | cellular component organization | 31 | 4 | 0.002 |
| **GO:0007568** | aging | 1 | 5 |  |
| **GO:0043473** | pigmentation | 13 | 5 | 0.440 |
| **GO:0006950** | response to stress | 73 | 16 | 0.000 |
| **GO:0006810** | transport | 55 | 19 | 0.050 |
| **GO:0055085** | transmembrane transport | 21 | 13 | 0.832 |
| **GO:0065007** | biological regulation | 20 | 6 | 0.153 |
| **GO:0065008** | regulation of biological quality | 7 | 3 |  |
| **GO:0006790** | sulfur metabolic process | 6 | 1 |  |
| **Molecular function** |  |  |  |  |
| **GO:0003824** | catalytic activity | 171 | 33 | 0.000 |
| **GO:0016491** | oxidoreductase activity | 42 | 9 | 0.005 |
| **GO:0016740** | transferase activity | 50 | 9 | 0.001 |
| **GO:0016741** | transferase activity, transferring one-carbon groups | 5 | 1 |  |
| **GO:0016757** | transferase activity, transferring glycosyl groups | 13 | 2 | 0.062 |
| **GO:0016772** | transferase activity, transferring phosphorus-containing groups | 24 | 6 | 0.059 |
| **GO:0016301** | kinase activity | 19 | 4 | 0.056 |
| **GO:0005488** | binding | 155 | 45 | 0.000 |
| **GO:0003677** | DNA binding | 22 | 5 | 0.051 |
| **GO:0008135** | translation factor activity, nucleic acid binding | 12 | 3 | 0.183 |
| **GO:0043167** | ion binding | 113 | 38 | 0.003 |
| **GO:0008092** | cytoskeletal protein binding | 5 | 3 |  |
| **GO:0019899** | enzyme binding | 4 | 1 |  |
| **GO:0005198** | structural molecule activity | 20 | 1 | 0.002 |
| **GO:0016874** | ligase activity | 13 | 4 | 0.265 |
| **GO:0016829** | lyase activity | 11 | 6 | 0.917 |
