# Supplementary material for: De Novo Assembled Wheat Transcriptomes Delineate Differentially Expressed Host Genes in Response to Leaf Rust Infection
Source: PLoS One. 2016 Feb 3;11(2):e0148453. doi: 10.1371/journal.pone.0148453 (PMC4739524; doi:10.1371/journal.pone.0148453)
Supplement: S6 Table — (DOC) [file pone.0148453.s019.doc]

**S6 Table**. List of the enriched GO terms showing the number of differentially upregulated contigs of S-PI and R-PI

| **GO-Term** | **Description** | **Upregulated in S-PI** | **Upregulated in R-PI** | **Enrichment P-value** |
| --- | --- | --- | --- | --- |
| **Cellular Component** |  |  |  |  |
| **GO:0005576** | extracellular region | 36 | 14 | 0.011 |
| **GO:0005623** | cell | 275 | 141 | 0.000 |
| **GO:0005622** | intracellular | 267 | 133 | 0.000 |
| **GO:0016020** | membrane | 42 | 28 | 0.322 |
| **GO:0030312** | external encapsulating structure | 17 | 8 | 0.163 |
| **GO:0005618** | cell wall | 17 | 8 | 0.163 |
| **GO:0005737** | cytoplasm | 253 | 113 | 0.000 |
| **GO:0009579** | thylakoid | 65 | 11 | 0.000 |
| **GO:0030529** | ribonucleoprotein complex | 18 | 9 | 0.190 |
| **GO:0043229** | intracellular organelle | 251 | 115 | 0.000 |
| **GO:0044446** | intracellular organelle part | 9 | 14 | 0.147 |
| **GO:0031974** | membrane-enclosed lumen | 7 | 13 | 0.085 |
| **GO:0032991** | macromolecular complex | 72 | 30 | 0.001 |
| **GO:0005840** | ribosome | 18 | 9 | 0.190 |
| **GO:0043234** | protein complex | 54 | 21 | 0.002 |
| **GO:0043226** | organelle | 251 | 116 | 0.000 |
| **GO:0031982** | vesicle | 52 | 14 | 0.000 |
| **GO:0043227** | membrane-bounded organelle | 250 | 112 | 0.000 |
| **GO:0005634** | nucleus | 43 | 32 | 0.577 |
| **GO:0005739** | mitochondrion | 64 | 39 | 0.099 |
| **GO:0005783** | endoplasmic reticulum | 22 | 8 | 0.034 |
| **GO:0005794** | Golgi apparatus | 15 | 7 | 0.185 |
| **GO:0009536** | plastid | 145 | 46 | 0.000 |
| **GO:0042579** | microbody | 2 | 8 |  |
| **GO:0005730** | nucleolus | 6 | 10 | 0.180 |
| **GO:0044428** | nuclear part | 8 | 14 | 0.093 |
| **GO:0005654** | nucleoplasm | 1 | 3 |  |
| **GO:0000229** | cytoplasmic chromosome | 0 | 1 |  |
| **Biological Process** |  |  |  |  |
| **GO:0000003** | reproduction | 15 | 17 | 0.407 |
| **GO:0008152** | metabolic process | 211 | 96 | 0.000 |
| **GO:0006807** | nitrogen compound metabolic process | 84 | 44 | 0.008 |
| **GO:0006139** | nucleobase, nucleoside, nucleotide and nucleic acid metabolic process | 23 | 19 | 0.937 |
| **GO:0006259** | DNA metabolic process | 11 | 12 | 0.541 |
| **GO:0016070** | RNA metabolic process | 3 | 5 |  |
| **GO:0009056** | catabolic process | 34 | 26 | 0.694 |
| **GO:0009058** | biosynthetic process | 94 | 45 | 0.001 |
| **GO:0043170** | macromolecule metabolic process | 77 | 43 | 0.025 |
| **GO:0010467** | gene expression | 20 | 22 | 0.393 |
| **GO:0006396** | RNA processing | 0 | 2 |  |
| **GO:0019538** | protein metabolic process | 68 | 33 | 0.007 |
| **GO:0043412** | biopolymer modification | 47 | 13 | 0.000 |
| **GO:0006082** | organic acid metabolic process | 22 | 11 | 0.147 |
| **GO:0006091** | generation of precursor metabolites and energy | 41 | 6 | 0.000 |
| **GO:0006790** | sulfur metabolic process | 7 | 3 |  |
| **GO:0015979** | photosynthesis | 53 | 5 | 0.000 |
| **GO:0051186** | cofactor metabolic process | 12 | 5 | 0.173 |
| **GO:0044238** | primary metabolic process | 144 | 76 | 0.001 |
| **GO:0005975** | carbohydrate metabolic process | 58 | 30 | 0.025 |
| **GO:0006629** | lipid metabolic process | 18 | 11 | 0.390 |
| **GO:0007010** | cytoskeleton organization | 1 | 2 |  |
| **GO:0000280** | nuclear division | 0 | 1 |  |
| **GO:0007049** | cell cycle | 3 | 6 |  |
| **GO:0007165** | signal transduction | 11 | 10 | 0.870 |
| **GO:0007155** | cell adhesion | 1 | 0 |  |
| **GO:0008219** | cell death | 6 | 4 |  |
| **GO:0016192** | vesicle-mediated transport | 6 | 5 | 0.979 |
| **GO:0051186** | cofactor metabolic process | 12 | 5 | 0.173 |
| **GO:0055085** | transmembrane transport | 30 | 9 | 0.004 |
| **GO:0006950** | response to stress | 85 | 38 | 0.001 |
| **GO:0044419** | interspecies interaction between organisms | 3 | 0 |  |
| **GO:0065007** | biological regulation | 20 | 13 | 0.455 |
| **GO:0007165** | signal transduction | 11 | 10 | 0.870 |
| **Molecular Function** |  |  |  |  |
| **GO:0003824** | catalytic activity | 195 | 86 | 0.000 |
| **GO:0016491** | oxidoreductase activity | 55 | 18 | 0.000 |
| **GO:0016740** | transferase activity | 80 | 33 | 0.000 |
| **GO:0016779** | nucleotidyltransferase activity | 6 | 9 | 0.270 |
| **GO:0016787** | hydrolase activity | 59 | 26 | 0.004 |
| **GO:0008233** | peptidase activity | 11 | 10 | 0.870 |
| **GO:0004518** | nuclease activity | 9 | 1 |  |
| **GO:0016874** | ligase activity | 10 | 13 | 0.303 |
| **GO:0003735** | structural constituent of ribosome | 6 | 5 | 0.979 |
| **GO:0022892** | substrate-specific transporter activity | 0 | 2 |  |
| **GO:0008565** | protein transporter activity | 0 | 2 |  |
| **GO:0005488** | binding | 173 | 88 | 0.000 |
| **GO:0003729** | mRNA binding | 0 | 2 |  |
| **GO:0008092** | cytoskeletal protein binding | 1 | 5 |  |
| **GO:0032182** | small conjugating protein binding | 0 | 1 |  |
| **GO:0045182** | translation regulator activity | 6 | 12 | 0.076 |
| **GO:0008135** | translation factor activity, nucleic acid binding | 6 | 12 | 0.076 |
| **GO:0043167** | ion binding | 141 | 64 | 0.000 |
